# The Ciliary Protein Ftm Is Required for Ventricular Wall and Septal Development

**DOI:** 10.1371/journal.pone.0057545

**Published:** 2013-02-28

**Authors:** Christoph Gerhardt, Johanna M. Lier, Stefanie Kuschel, Ulrich Rüther

**Affiliations:** Institute for Animal Developmental and Molecular Biology, Heinrich Heine University, Düsseldorf, Germany; Northwestern University, United States of America

## Abstract

Ventricular septal defects (VSDs) are the most common congenital heart defects in humans. Despite several studies of the molecular mechanisms involved in ventricular septum (VS) development, very little is known about VS-forming signaling. We observed perimembranous and muscular VSDs in *Fantom* (*Ftm*)-negative mice. Since Ftm is a ciliary protein, we investigated presence and function of cilia in murine hearts. Primary cilia could be detected at distinct positions in atria and ventricles at embryonic days (E) 10.5–12.5. The loss of Ftm leads to shortened cilia and a reduced proliferation in distinct atrial and ventricular ciliary regions at E11.5. Consequently, wall thickness is diminished in these areas. We suggest that ventricular proliferation is regulated by cilia-mediated Sonic hedgehog (Shh) and platelet-derived growth factor receptor α (Pdgfrα) signaling. Accordingly, we propose that primary cilia govern the cardiac proliferation which is essential for proper atrial and ventricular wall development and hence for the fully outgrowth of the VS. Thus, our study suggests ciliopathy as a cause of VSDs.

## Introduction

One of 100 newborns suffers from a congenital heart defect [1]. Among these human congenital cardiac diseases ventricular septal defects (VSDs) are the most common [2], [3] and occur in approximately 1 of 1000 births [4]. The most prevalent VSD subtype is the perimembranous VSD [5], [6] which is characterized by the loss of the membranous part of the ventricular septum (VS) and a defect in the development of a second part of the VS - the muscular septum. Interestingly, the membranous VS does not start to grow before the muscular VS generation has been finished [7] indicating that membranous VS development is probably initiated by an interaction of the inlet muscular VS and the atrioventricular endocardial cushion cells (ECCs) [8], [9]. The membranous VS arises solely from the ECCs and not from the muscular VS [4]. Although the molecular background of muscular VS development is only poorly understood [10], [11], two different hypothesis have been debated for its formation and outgrowth. The first theory describes VS generation as an active process of cell growth in the apical region of the muscular septum [12], [13], while the second ascribes muscular septal length gain to a passive process based on the increase of the ventricular cavities. According to this hypothesis, the formation of the muscular septum is carried out by proliferation of cells at distinct regions in the left and right ventricle [14]–[17], so that it consists of cardiomyocytes with both left-ventricular and right-ventricular identities [11], [18], [19].

*Ftm (alias Rpgrip1l)*-negative mice display abnormal heart development particularly a VSD and suffer from a dysfunction of primary cilia [20] indicating a potential relation between heart formation and ciliary action. The Ftm protein is localised at the base of cilia [20] and appears to be present at every cilium. The fact that mutations of *FTM* in humans were already found in ciliopathies like Meckel-Gruber syndrome, Joubert syndrome and nephronophthisis [21], [22] accentuates the importance of this gene in human development.

Primary cilia are hairlike, 1–15 µm long protrusions on most vertebrate cells. They function as the cells “antenna” receiving and mediating signals from the environment. These signals, in turn, control important cellular processes like proliferation, apoptosis, migration, differentiation and cell cycle regulation [23]. Consequently, defective primary cilia provoke severe human diseases [24]. Several signaling pathways are thought to be associated with cilia, including Sonic hedgehog (Shh), platelet-derived growth factor receptor α (Pdgfrα) as well as canonical and non-canonical Wnt signaling [25]–[35]. While the connection between cilia and Wnt signaling has been frequently discussed and remains the subject of fierce debate [34]–[36], it is well-known that Shh and Pdgfrα signaling can be mediated by cilia [25]–[27], [29], [30], [35].

Shh is a member of the Hedgehog (Hh) family of evolutionary conserved signaling molecules and binds to its receptor Patched (Ptc) which in vertebrates is localized in the ciliary membrane and regulates the activity of Smoothened (Smo), a seven-transmembrane receptor. Recruited to the cilium active Smo invokes Glioblastoma (Gli) transcription factors. In vertebrates three Gli isoforms exist – Gli1, 2 and 3. They regulate the expression of Shh target genes like for example *Ptc1* and thereby cell differentiation, proliferation, survival and growth [37], [38]. Gli1 functions as a constitutive activator [39], [40], whereas Gli2 and Gli3 have a C-terminal transcriptional activator domain and a N-terminal transcriptional repressor domain [41]. Full-length Gli3 (Gli3-190) protein can be transformed into a transcriptional activator (Gli3-A) most likely by modifications [42], [43]. Importantly, the full-length protein can be proteolytically processed into a transcriptional repressor (Gli3-R, also known as Gli3-83) [44]. The ratio of activator and repressor forms controls cellular processes dependend on Shh signaling.

Signaling by Pdgfrα relates also to cilia [29]. Pdgfrα is localized to cilia and becomes dimerized and phosphorylated after being bound by its ligand Pdgf-AA which also functions as a dimer. Activated Pdgf receptors regulate essential cell processes like proliferation, anti-apoptosis, migration, differentiation, actin reorganization and cell growth [45]–[47]. Stimulation of Pdgfrα drives the activation of signal transduction through the Mek1/2-Erk1/2 and Akt/PKB pathways mediated by primary cilia, whereas Pdgfrα signaling gets blocked in the absence of cilia [29].

We used *Ftm*-deficient mice to investigate whether cardiac cilia are functionally involved in heart development, especially in VS formation. Furthermore, we analysed which signals are mediated by these cilia. We were able to identify components of Shh and Pdgfrα signaling pathways in or at ventricular cilia giving evidence that these signals are cilia-mediated in embryonic murine hearts. According to ciliary dysfunction caused by Ftm deficiency [21], [48], [49], Shh and Pdfgrα signaling are downregulated in *Ftm*-negative ventricles. We propose these signaling defects as the cause of reduced ventricular cell proliferation that in turn results in diminished ventricular wall thickness and VSDs.

## Materials and Methods

### Ethics Statement and Animal Husbandry

All mice (Mus musculus) used in this study were on the C3H background and kept under standard housing conditions with a 12/12 hours dark-light cycle and with food and water ad libitum. All experiments were performed in accordance with the relevant national guidelines for the Care and Use of Laboratory Animals, with approval from the authority for animal work at the Heinrich Heine University (Permit Number: O18/99). Generation of *Ftm* mutant mice was designed and carried out as described [20].

### Antibodies

We used primary antibodies to actin (Sigma #A2066), Arl13b (Proteintech #17711-1-AP), Gapdh (Sigma #G8795), acetylated α-tubulin (Sigma #T6793), γ-tubulin (Sigma #T6557), detyrosinated tubulin (Millipore #AB3201), BrdU (Developmental Studies Hybridoma Bank #G3G4), Pdgfrα (Santa Cruz #sc-338), pericentrin (Covance #PRB-432C), pMek1/2 (Cell Signaling Technology #9121), Gli3 (kindly gift of B. Wang), Gli3 (R&D systems #AF3690), ErbB3 (Santa Cruz #sc-285), DDR2 (kindly gift of E.C. Goldsmith) and Tropomyosin (AbD Serotec #9200-0504). The creation of polyclonal antibodies against Ftm was delineated formerly [20]. Polyclonal antibodies to Gli3-190 were generated by immunizing rabbits with a His-Gli3 fusion protein encompassing the Gli3-C-terminal region (3473–4806 bp) by Pineda antibody services. Antibodies were affinity-purified with the antigen coupled to Ni-NTA agarose (Qiagen #30230).

### Apoptosis Studies

Apoptotic nuclei were labeled *in situ* by the TdT-mediated dUTP-biotin nick end labeling (TUNEL) method [50] using Apop Taq Plus Peroxidase *in situ* Apoptosis Kit (Millipore #S7101) and following manufacturer’s instructions.

### Genotyping

Genotyping of the mice was performed as previously described [20].

### Histochemistry

Histochemical stainings were performed as described [20].

### Histology and Paraffin Embedding

Embryos were dissected and fixed in 4% paraformaldehyde (PFA) overnight at 4°C. Then they were serially dehydrated using ethanol, embedded in paraffin and sectioned (12 µm). Afterwards, sections were stained with hematoxylin and eosin or used for *in situ* hybridisation.

### Immunofluorescence

Embryos were fixed in 4% PFA and incubated in 30% sucrose (in PBS) overnight at 4°C. Next day they were embedded in Tissue-Tek O.C.T. compound (Sakura Finetechnical #4583) and then stored at −80°C. Transverse cryostat sections (7 µm in thickness) were prepared, washed with PBS and permeabilized with PBS/0.5% Triton-X-100. Blocking was performed with 10% FCS in PBS/0.1% Triton-X-100. The sections then were incubated with the primary antibodies diluted in blocking solution overnight at 4°C. After three washing steps, they underwent an incubation in the secondary antibody (diluted in blocking solution) for 2 hours and then were washed again. Finally, they were embedded in Mowiol containing DAPI (Merck #1.24653).

### *In situ* Hybridisation

*In situ* hybridisation on paraffin sections were performed as previously described [51].

### Proliferation Studies

Mice received an intraperitoneal injection of 10 µl BrdU (Sigma #B5002-1G) per g body weight 2 hours before they were killed. After killing embryos were dissected and embedded in Tissue-Tek O.C.T. compound (Sakura Finetechnical #4583) as described before. Cryosections were undergone BrdU immunohistochemical stainings like described before with the exception of two additionally steps after the first washings: These steps include incubation in 2 N HCl for 10 minutes at 37°C and then in 50% formamide/2×SSC for 45 minutes at 65°C. Anti-BrdU (Developmental Studies Hybridoma Bank #G3G4) antibody was used as primary antibody.

### Real-time PCR Analysis

Atrial and ventricular RNA was isolated by using RNeasy Kit (Qiagen #74104) and RNase-Free DNase Set (Qiagen #79254). Isolated RNA was converted into cDNA by utilising Expand Reverse Transcriptase (Roche #11785826001). Quantitative Real-time PCR was carried out by employing a Step One Real-Time PCR System Thermal Cycling Block (Applied Biosystems #4376357) and the TaqMan Universal PCR Master Mix, No AmpErase UNG (Applied Biosystems #4324020). The following primer/TaqMan probe sets were used: *Gapdh* (Assay ID: Mm99999915_g1), *Ptc1* (Assay ID: Mm00970977_m1) and *Hif1α* (Assay ID: Mm00468878_m1). Real-time PCR was carried out with 50 ng of cardiac cDNA of each sample in triplicate reactions in a 20 µl volume containing 100 nM primers and 50 nM probe. Cycling conditions were 50°C for 2 minutes and 95°C for 10 minutes, followed by a 40-cycle amplification of 95°C for 15 seconds and 60°C for 1 minute. The analysis of real-time data was performed by using included StepOne Software version 2.0.

### Semiquantitative PCR Analysis

RNeasy Kit (Qiagen #74104) was used to isolate mRNA from pooled embryonic hearts (E11.5). Reverse transcription was carried out by utilising High Capacity RNA-to-cDNA Master Mix (Applied Biosystems #4390777). The sets of primers were as following: *Hprt*: 5′-CAC AGG ACT AGA ACA CCT GC (forward), 3′-GCT GGT GAA AAG GAC CTC T (reverse); *Cyclin E*: 5′-CTG GCT GAA TGT TTA TGT CC (forward), 3′-TCT TTG CTT GGG CTT TGT CC (reverse); *p27*∶5′-AAC CTC TTC GGC CCG GTG GAC CAC (forward), 3′-GTC TGC TCC ACA GAA CCG GCA TTT (reverse).

### Statistical Data

To compare percentage of proliferating cells and percentage of apoptotic cells in wild-type and *Ftm*-mutant hearts, we counted BrdU or TUNEL marked cells and total number of cells (DAPI-marked) in distinct regions on ten different, transverse sections per heart, averaged over them and related them to each other. All heart chambers could be analysed on every section. Thereby, we differentiated between ciliary, former ciliary and non-ciliary regions.

To contrast wild-type with *Ftm*-negative cardiac wall thickness, the measurements of wall thickness were performed at distinct regions on ten different, transverse sections per heart. All four heart chambers were uncovered on every section. The measured values per heart were averaged.

Data are presented as mean ± standard deviation. Student’s *t* test was performed to compare percentage of proliferating cells, cardiac wall thickness, RNA-expression levels and percentage of apoptotic cells in wild-type and *Ftm*-mutant hearts by using Graphpad and Microsoft Excel. A *p* value <0.05 was considered to be statistically significant (one asterisk), a *p* value <0.01 was regarded as statistically very significant (two asterisks) and a *p* value <0.001 was accounted statistically high significant (three asterisks).

### Western Blotting

Western blot studies were done essentially as described using anti-Gli3 antibody or anti-pMek1/2 antibody [44]. Anti-actin antibody and anti-Gapdh antibody were used as control for loading. Visualising of Gli3, pMek1/2, actin and Gapdh bands was realised by LAS-4000 mini (Fujifilm #8692184). Bands were measured in intensity using Adobe Photoshop 7.0.

## Results

### Ftm-negative, Murine Embryos Display Muscular and Perimembranous Ventricular Septal Defects

**Figure 1:**
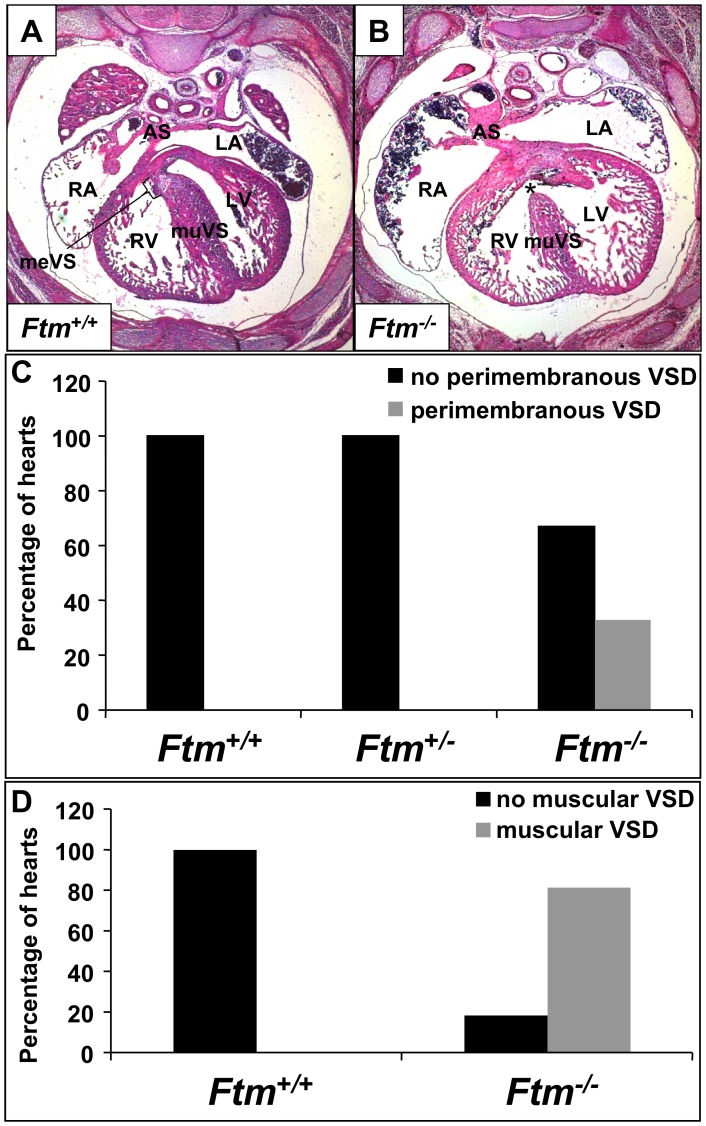
*Ftm*-deficient murine embryos show perimembranous and muscular ventricular septal defects. (A, B) Hematoxylin and Eosin stainings at E14.5 on transverse heart sections. (A) In wild-type mouse embryos, the ventricular septum consists of a muscular part (muVS) and a membranous part (meVS). (B) In *Ftm* ^−/−^ embryos, the muscular VS displays a shorter and thinner shape and the membranous VS is missing (indicated by the asterisk) representing a perimembranous ventricular septal defect. (C) While in *Ftm* ^+/+^ (n = 23) and *Ftm* ^+/−^ mice (n = 21) the heart develops normally, 33% of *Ftm* ^−/−^ mice (n = 27) show perimembranous ventricular septal defects. This statistics is based on investigations of mice at E13.5, E14.5, E15.5, E16.5 and E17.5. (D) *Ftm* ^+/+^ mouse embryos (n = 23) do not suffer from muscular ventricular septal defects. 81.5% of all analyzed *Ftm* ^−/−^ embryos (n = 27) display muscular ventricular septal defects. Embryos at E13.5, E14.5, E15.5, E16.5 and E17.5 were examined in this context. LA, left atrium; RA, right atrium; LV, left ventricle; RV, right ventricle; meVS, membranous ventricular septum; muVS, muscular ventricular septum; AS, atrial septum.

33% of all analysed *Ftm*-homozygous mutant embryos (9 of 27 embryos) show perimembranous VSDs marked by the combination of a significantly thinner muscular part of the VS and the absence of the membranous part of the VS (Figure 1B, C), while none of the *Ftm*-heterozygous mutants exhibits an abnormal heart phenotype (Figure 1C). We measured the length and thickness of ventricular and atrial septa in *Ftm* ^+/+^ and *Ftm* ^−/−^ hearts, respectively, and found out that the atrial septum (AS) displays no differences between the wild-type and *Ftm*-negative state (data not shown). Furthermore, we did not observe any morphological AS abnormalities. In contrast to the atria, *Ftm*-deficient ventricles display defects, but the length measurements do not reflect a significant alteration at different embryonic days (Figure S1A, D, G, J, M). This is due to the fact that the frequency of perimembranous VSDs in the absence of Ftm is too low during embryonic development (Figure S1B, E, H, K, N). At E13.5 40% of all analyzed *Ftm* ^−/−^ mouse embryos (2 of 5) suffer from perimembranous VSDs, at E14.5 50% (3 of 6), at E15.5 67% (2 of 3), at E16.5 0% (none of 4) and at E17.5 22% (2 of 9). Compared to the length measurements, the width of *Ftm*-negative VS is significantly reduced (Figure S1A, D, G, J, M) characterizing a muscular VSD. 81, 5% of all analyzed *Ftm* ^−/−^ embryos (22 of 27 embryos) suffer from muscular VSDs (Figure 1D). The reduction of muscular VS width is significant at all analyzed embryonic days from E13.5 to E17.5 (Figure S1A, D, G, J, M), since the frequency of muscular VSDs is high in all embryonic stages (Figure S1C, F, I, L, O). At E13.5 muscular VSDs can be observed in 80% of all analysed *Ftm*-negative embryos (4 of 5), at E14.5 in 83% (5 of 6), at E15.5 in 100% (3 of 3), at E16.5 in 50% (2 of 4) and at E17.5 in 89% (8 of 9). These data indicate that the muscular VS defect takes place in a high frequency even if the loss of the membranous VS occurs only in a minority of all *Ftm* ^−/−^ embryos leading to the conclusion that muscular VS development is severly disturbed in most *Ftm*-deficient embryos.

To test if the appearance of perimembranous VSDs correlates with other defects of *Ftm*-deficient mice, we looked for the entire phenotype of all analyzed mice. Comparing the different phenotypes, there seems to be no correlation between the occurrence of perimembranous VSDs and other abnormalities (Table S1).

#### Cilia are absent from the VS in E10.5 to E12.5 murine hearts

**Figure 2:**
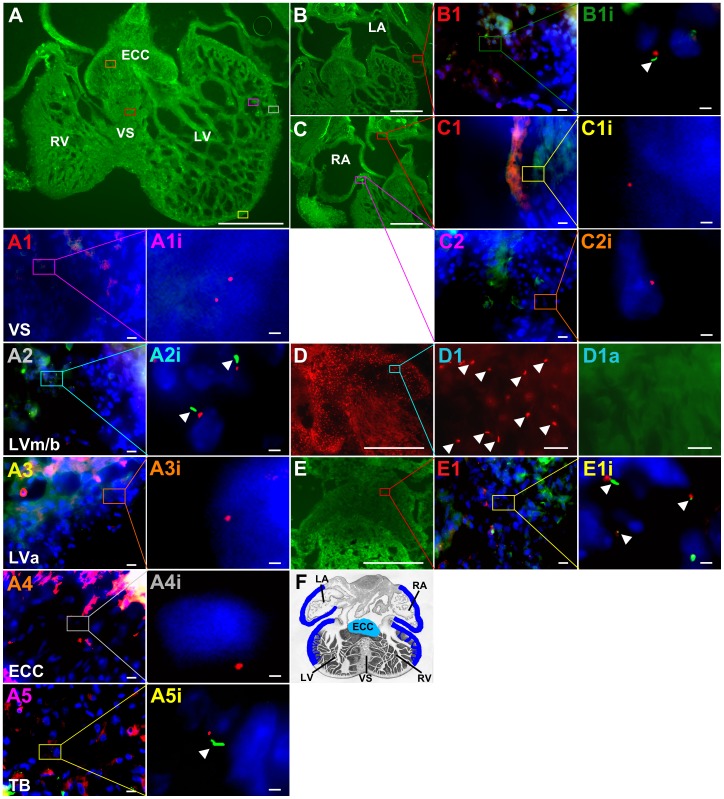
Distribution of primary cilia in murine embryonic hearts. (A, B, C, D, E, A1−5, B1, C1+2, D1, E1, A1i−A5i, B1i, C1i, C2i, D1a, E1i) Immunofluorescence on transverse heart sections at E12.5. (A, B, C) Tubulin cytoskeleton of cardiac cells is stained in green by acetylated α-tubulin resulting in a total view of ventricles (A) and atria (B, C). Cilia are stained in green by acetylated α-tubulin, basal bodies in red by pericentrin and cell nuclei in blue by DAPI (A1−5, B1, C1+2, A1i−A5i, B1i, C1i, C2i). Scale bars (in white) represent a length of 0.5 mm (A, B, C, D, E), 10 µm (A1−5, B1, C1+2, D1, D1a, E1) or 2 µm (A1i−A5i, B1i, C1i, C2i, E1i). (A, B, C, D, E) Coloured squares mark cardiac regions which are presented magnified in A1−5, B1, C1+2, D1+1a and E1. (A1−A5, B1, C1+2, E1) Coloured squares mark cardiac regions which are shown magnified in A1i-A5i, B1i, C1i, C2i and E1i. (A, B, C, D, E, A1−A5, B1, C1, C2, D1, D1a, E1, A1i−A5i, B1i, C1i+2i, E1i) The colour of the square correlates with the colour of the number of the magnified figures. (D) Arl13b is stained in red resulting in a total view of ECCs. (D1) Arl13b staining reveals ciliary presence on ECCs, while these cilia cannot be detected by staining acetylated α-tubulin in green (D1a). (E) Tubulin cytoskeleton of cardiac cells is stained in green by detyrosinated tubulin resulting in a total view of the ECCs. (E1, E1i) Cilia are stained in green by detyrosinated tubulin, basal bodies in red by γ-tubulin and cell nuclei in blue by DAPI. (F) Schematic illustration of ciliary distribution in embryonic mouse hearts. We found cilia exclusively at E10.5–12.5 and solely in distinct ventricular and atrial regions (blue lines) and on ECCs (turquoise staining). ECC, endocardial cushion cells; LA, left atrium; RA, right atrium; LV, left ventricle; LVa, left ventricle apical; LVm/b left ventricle medial/basal; RV, right ventricle; TB, trabecular formations; VS, ventricular septum.

Since Ftm is a cilia-associated protein [20], we assumed that the cause of heart phenotype in *Ftm*-negative mice could be a ciliary dysfunction, although never before a ciliopathy was regarded as the elicitor of VSDs. The pre-condition for this assumption is the presence of cilia in murine hearts. In previous studies, cardiac cilia were detected in mice [52], [53], but it was not mentioned if the VS is ciliated. We observed monocilia in a very distinct spatial distribution from E10.5 to E12.5 (Figure 2), but could never detect any cilia on VS cells (Figure 2A1, A1i). Furthermore, we could not demonstrate the presence of cilia on those ventricular cells which are close to the base of the muscular VS (Figure 2A3, A3i). Interestingly, cilia on ECCs were hardly detectable by visualizing acetylated α-tubulin (Figure 2D1a). Instead, the detection of Arl13b reveals ciliary presence also on the surface of these cells (Figure 2D1). Since acetylated α-tubulin serves as a marker for the ciliary axoneme, we tested if ECC cilia lack axonemes. Therefore, we used an antibody to detyrosinated tubulin which is another tubulin modification indicative for the ciliary axoneme [54]. Detyrosinated tubulin was detected on ECCs (Figure 2E, E1, E1i) demonstrating that ECC cilia exhibit an axoneme. To proof if cilia can be observed on VS cells by using other ciliary marker instead of labelling acetylated α-tubulin, we performed antibody stainings with an anti-Arl13b antibody. But even by marking Arl13b, we could not detect cilia on VS cells (Figure S2) or in other non-ciliary regions. Marking different cardiac cell types, we found cardiac cilia poking out of myocardial and endocardial cells (Figure S3A, B), but not from cardiac fibroblasts (Figure S3C).

#### Cardiac cilia are shortened in Ftm-homozygous mutant mice

**Figure 3:**
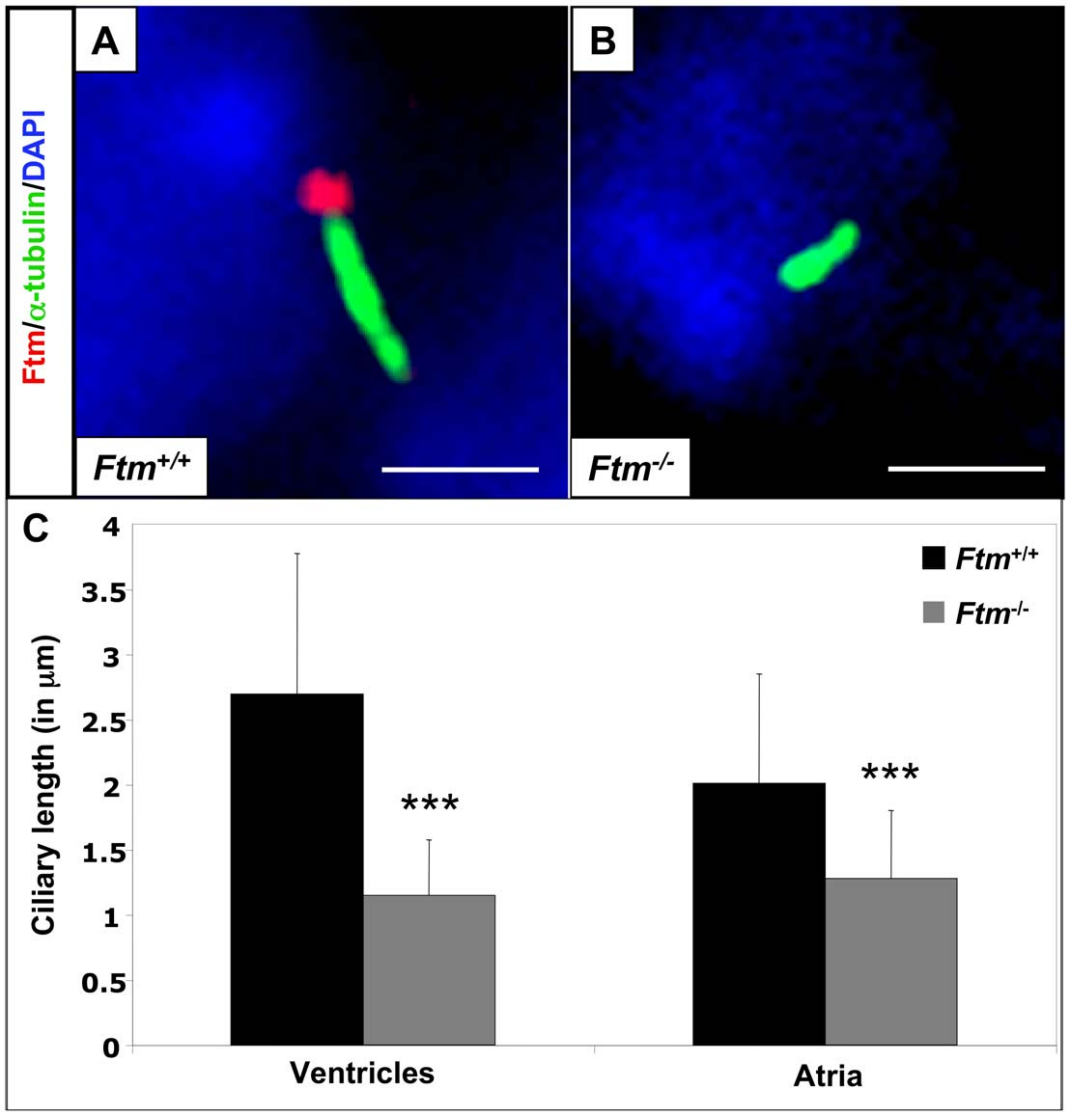
Loss of Ftm leads to shorter cardiac cilia. (A, B) Immunofluorescence on transverse heart sections at E11.5. Cilia are stained in green by acetylated α-tubulin and cell nuclei in blue by DAPI. Scale bars (in white) represent a length of 2 µm. Ftm is localised at cardiac cilia (A), but is absent from *Ftm*-negative cilia (B). (C) Comparison of wild-type and *Ftm*-deficient ciliary length in ventricles and atria (n = 50 cilia, respectively). *Ftm*-negative cilia are significantly shorter in both ventricles (p = 3.41E−12) and atria (p = 2.79E−06).

We previously showed that Ftm is present at the base of cilia in cell culture [20] and others observed Ftm at cilia of murine eyes and brains [48], but nothing is known about the localisation of Ftm at cardiac cilia. Consequently, we looked for Ftm in wild-type hearts. The staining of Ftm and acetylated α-tubulin (indicative for the ciliary axoneme) in combination with the partly overlapping staining of Ftm and γ-tubulin (basal body marker) reveals that Ftm is located at the base of atrial and ventricular cilia (Figure 3A; Figure S4A). Meanwhile, Ftm is completely missing in *Ftm*-negative embryos (Figure 3B; Figure S4B). Since the loss of Ftm in some cilia leads to a change in ciliary morphology (e.g. nodal cilia; [20]) and since the alteration of ciliary length leads to ciliary dysfunction [27],[55]–[57], we analysed the length of *Ftm*-homozygous mutant, cardiac cilia at E11.5. Cilia of *Ftm*-deficient ventricles and atria are clearly shorter than in the wild-type (Figure 3C) arguing for a possible ciliary dysfunction in those hearts. Thus, Ftm is necessary for regulating the length of cardiac cilia.

#### Less proliferation at ciliary regions in Ftm-deficient hearts

**Figure 4:**
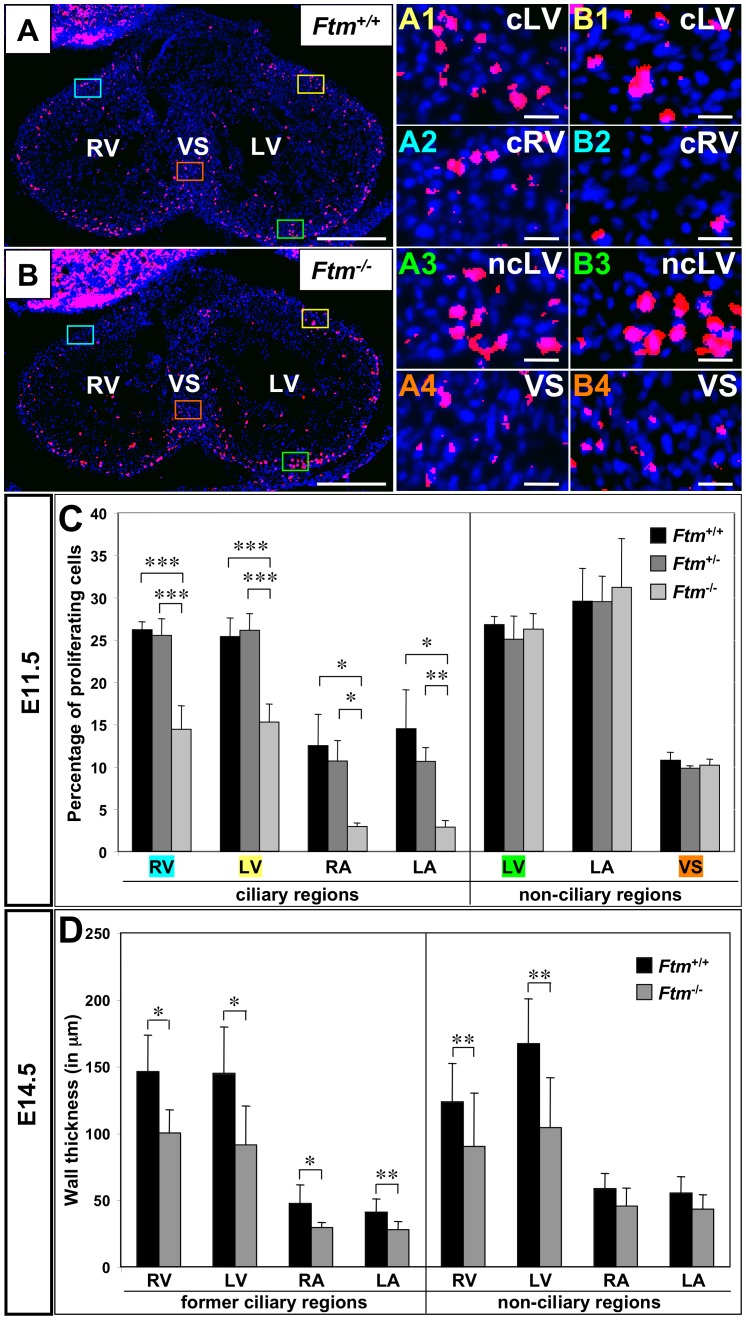
Reduced proliferation in ciliary regions of *Ftm*-deficient murine hearts and thickness decrease of *Ftm*-negative walls. (A, B, A1–4, B1–4) Immunofluorescence on transverse ventricular sections at E11.5. Dividing cells (red staining) are marked by BrdU and cell nuclei (blue staining) by DAPI. Scale bars (in white) represent a length of 0.5 mm (A, B) or 20 µm (A1–4, B1–4). (A, B) Coloured squares mark cardiac regions which are presented magnified in A1–4 and B1–4, respectively. The colour of the square correlates with the colour of the number of the magnified figures. (C) Proliferation rate is determined by the relation of dividing (BrdU-marked) cells to the number of all cells in this heart region at E11.5 (*Ftm* ^+/+^: n = 6; *Ftm* ^+/−^: n = 11; *Ftm* ^−/−^: n = 5). There is significantly less proliferation in the ciliary regions of ventricles and atria compared to non-ciliary regions. (D) Cardiac wall thickness measurements of wild-type (n = 6) and *Ftm*-deficient (n = 6) atria and ventricles in former ciliary and non-ciliary regions at E14.5. Walls are significantly thinner in all former ciliary regions. Additionally, ventricular, non-ciliary regions show a reduction in wall thickness, while atrial, non-ciliary regions do not differ significantly. LA, left atrium; RA, right atrium; LV, left ventricle; cLV, ciliary region of the left ventricle; ncLV, non-ciliary region of the left ventricle; RV, right ventricle; ciliary region of the right ventricle; VS, ventricular septum.

The VS does not fully grow out in *Ftm*-negative mice (Figure 1B). From other tissue and cell culture experiments, it is known that monocilia mediate proliferative and apoptotic signals [23]. Since ventricular cilia appear at the time, when the muscular VS is growing out [58], and at those regions, where the proliferation of cells effects the outgrowth of the VS [11], we investigated proliferation and apoptosis via bromodeoxyuridine (BrdU) staining to determine the rate of proliferation and by means of TdT-mediated dUTP-biotin nick end labeling (TUNEL) staining to look for cell death at E11.5. Whereas the apoptosis study was inconspicuous (Figure S5A), there were differences in the proliferation rate between wild-type, *Ftm*-heterozygous and *Ftm*-homozygous mutant hearts. The proliferation in all *Ftm*-negative, ciliary areas in ventricles and atria was significantly diminished, while no proliferation differences could be observed in non-ciliary regions (Figure 4B1–B4, C). So in embryonic hearts, cilia seem to be necessary to mediate proliferative signals which in turn are responsible for a part of cardiac cell proliferation. Consequently, when cilia are absent at a later point of time, the rate of proliferation of wild-type, *Ftm*-heterozygous and *Ftm*-homozygous mutant embryos should not differ significantly. Performing the same proliferation assays in E14.5 hearts, we found that, indeed, the proliferation in all areas, which were investigated, was similar in wild-type and *Ftm*-deficient hearts (Figure S5B). The diminished proliferation in *Ftm*-homozygous mutant hearts is in agreement with the results of semiquantitative Reverse transcription-PCRs. These experiments uncovered a change of expression levels of *cyclin E* and *p27* that are involved in cell cycle regulation and proliferation (Figure S6) substantiating suspicion of a proliferation defect in *Ftm*-deficient hearts.

#### Reduction of wall thickness in Ftm-negative hearts

Suggesting ciliary dysfunction is responsible for the decline of cell number in *Ftm*-deficient ventricular walls, we supposed that ventricular walls could be thinner in *Ftm*-negative than in wild-type mice. Analysis of ventricular wall sizes reveals a decrease of wall thickness in all regions of *Ftm*-negative hearts at E14.5, where cilia were present at E11.5 (Figure 4D). Furthermore, ventricular walls of *Ftm*-deficient mice without ciliary presence at any time are significantly thinner than those of wild-type mice at E14.5 (Figure 4D). These walls reside close to the base of the muscular VS. We also detected a reduction of wall thickness in all ciliary atrial areas, but not in non-ciliary regions of the atria (Figure 4D). Remarkably, 100% of all analyzed *Ftm*-negative embryos (6 of 6) display a decreased wall thickness in atria and ventricles (data not shown).

#### Shh and Pdgfrα signals are downregulated in Ftm-deficient hearts

**Figure 5:**
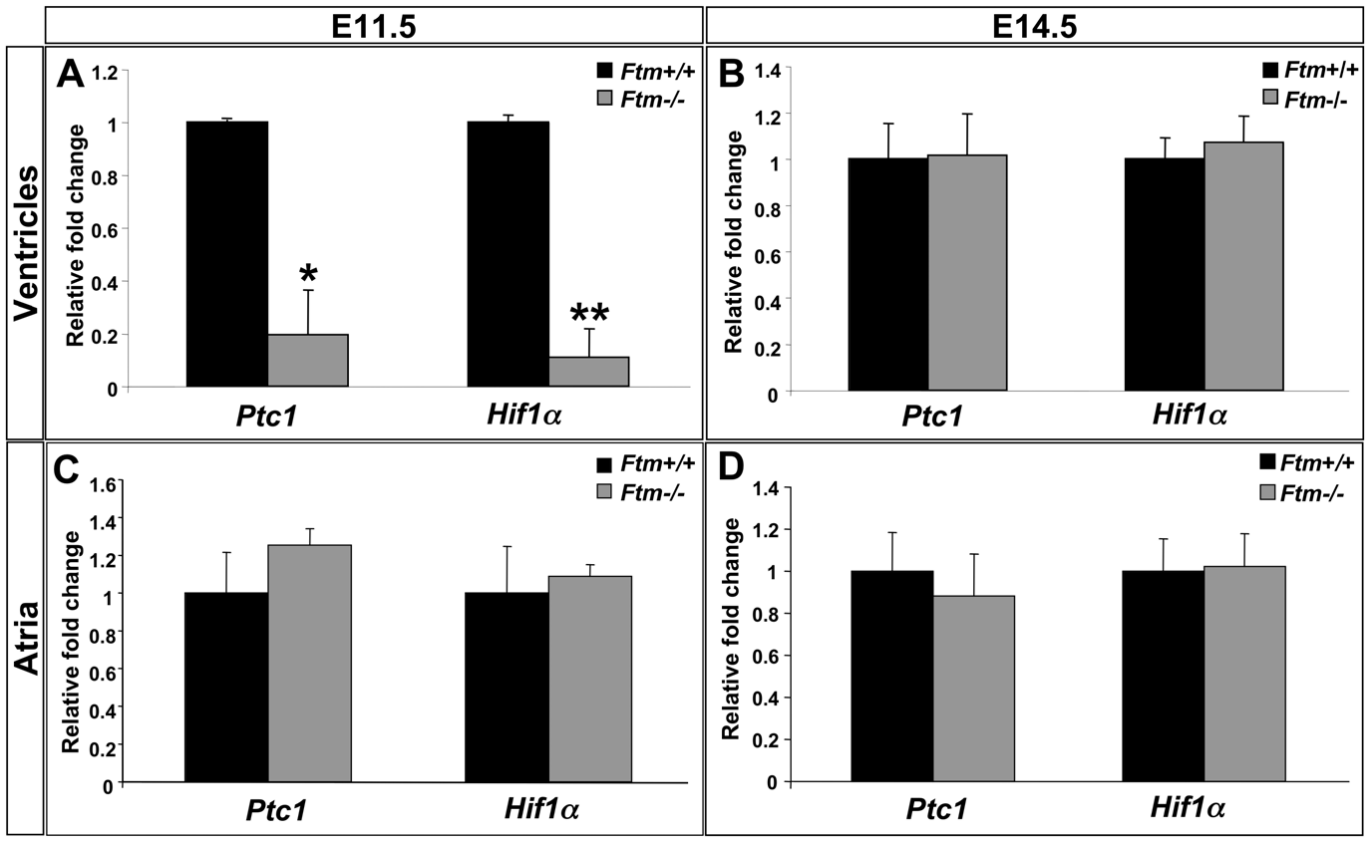
Shh and Pdgfrα signals are downregulated in *Ftm*-negative murine hearts. (A–D) Real-time PCR analysis of wild-type and *Ftm*-deficient ventricular (A, B) and atrial tissue (C, D) at E11.5 (A, C) and E14.5 (B, D). (A) Shh target gene expression of *Ptc1* and Pdgfrα target gene expression of *Hif1α* are significantly downregulated in E11.5 *Ftm* ^−/−^ ventricles (n = 3, respectively; *Ptc1*: p = 0.013; *Hif1α*: p = 0.005). (B) At E14.5, both signaling pathways are unaffected in *Ftm*-negative ventricles (n = 6, respectively). (C, D) In *Ftm* ^−/−^ atria, Shh and Pdgfrα signaling are not significantly altered at E11.5 (n = 6 atria, respectively; C) and at E14.5 (n = 6 atria, respectively; D).

Primary cilia are mediators of signaling pathways, which activate certain cellular processes. To elucidate, which signals are indispensable for cilia-controlled, cardiac proliferation, we looked for target gene expression of signaling pathways from which is known that they are mediated by cilia [25], [35]. Thereby, *Patched1* (*Ptc1*) is used as target gene of Shh signaling [59] and *Hypoxia-inducible factor 1, α subunit* (*Hif1α*) of Pdgfrα signaling [60]. Gene expression studies were performed at a ciliary as well as at a non-ciliary period (E11.5 and E14.5, respectively) and the hearts got subdivided into the ventricular and the atrial part to differ between ventricular and atrial ciliary signal mediation. At E11.5, *Ftm*-deficient ventricles show a significant downregulation of Shh and Pdgfrα signaling (Figure 5A), but these signaling pathways are unaltered in *Ftm*-negative atria (Figure 5C). At the non-ciliary stage E14.5, we do not see expression alterations of the analysed target genes in the *Ftm* ^−/−^ state (Figure 5B,D). Taken together, these results show a downregulation of signaling pathways in *Ftm*-homozygous mutant hearts at E11.5, but no differences at the non-ciliary stage E14.5.

#### Most likely Shh signaling acts upstream of Pdgfrα signaling in ventricular cilia and is disturbed in *Ftm*-deficient hearts owing to a Gli3 processing defect

**Figure 6:**
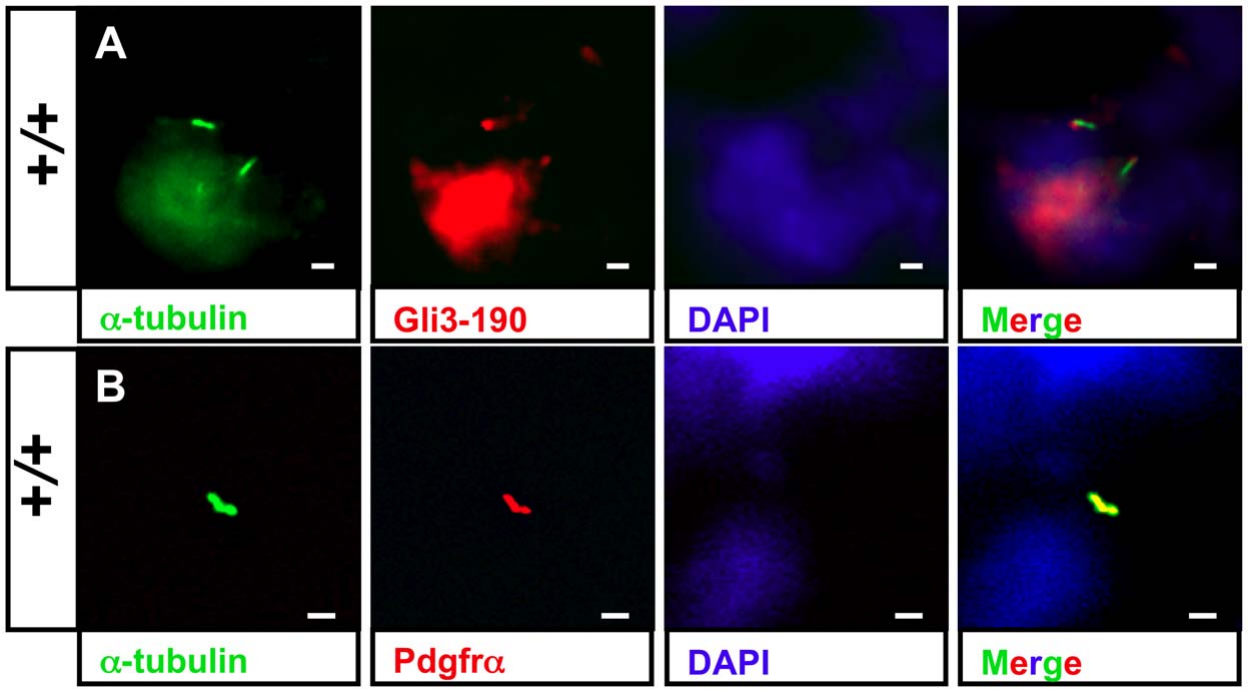
Shh and Pdgfrα signaling components localize at cardiac cilia. (A+B) Immunofluorescence on transverse heart sections at E11.5. Cilia are stained in green by acetylated α-tubulin and cell nuclei in blue by DAPI. Scale bars (in white) represent a length of 2 µm. (A) Ciliary Gli3-190 localisation (red staining) in wild-type ventricles demonstrates that Shh signaling is transduced by ventricular cilia. (B) Pdgfrα (red staining) is distributed along cardiac cilia in wild-type ventricles.

**Figure 7:**
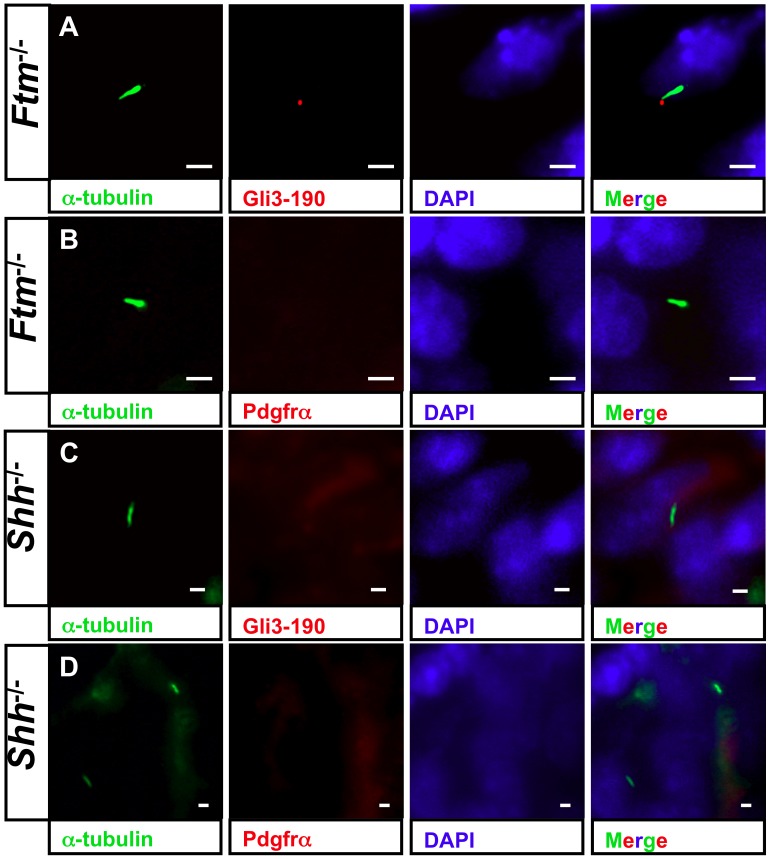
Shh signaling acts upstream of Pdgfrα signals in cardiac cilia. (A–D) Immunofluorescence on transverse heart sections at E11.5. Cilia are stained in green by acetylated α-tubulin and cell nuclei in blue by DAPI. Scale bars (in white) represent a length of 2 µm. (A) Gli3-190 (red staining) still shows a ciliary localisation in ventricular *Ftm* ^−/−^ cilia. (B) Pdgfrα (red staining) is absent in cilia of *Ftm*-deficient ventricles. (C) Gli3-190 protein is not observed at cilia of *Shh* ^−/−^ ventricles. (D) Pdgfrα cannot be detected in cilia of *Shh*-negative ventricles.

To elucidate if these signaling pathways are mediated by cardiac cilia, we performed immunofluorescence stainings of proteins which are essential for Shh and Pdgfrα signaling, respectively. The Shh signaling mediator Gli3-190 [41] can be clearly observed at the base of ventricular cilia (Figure 6A, Figure S7A, B) and Pdgfrα is present all along ventricular cilia (Figure 6B). We could neither detect Gli3-190 nor Pdgfrα at E11.5 atrial cilia (data not shown). This indicates that both signaling pathways are mediated by cilia in ventricles but not in atria at E11.5. Since we already detected a downregulation of Shh and Pdgfrα signaling in *Ftm*-deficient hearts via qRT-PCR, we looked for Gli3-190 and Pdgfrα localisation in *Ftm*-negative cardiac cilia. In these cilia, Gli3-190 is still present (Figure 7A), while Pdgfrα gets lost in ventricular cilia (Figure 7B). These results let assume that Pdgfrα signaling acts downstream of Shh signaling. To confirm this hypothesis, we investigated Gli3-190 and Pdgfrα localisation at *Shh*-deficient cilia in the heart. Gli3-190 and Pdgfrα are absent in ventricular cilia (Figure 7C, D) resulting in the conclusion that Pdgfrα signaling functions downstream of Shh signaling in ventricular cilia. The dependency of ventricular Pdgfrα signaling on Shh signaling is confirmed by a smaller amount of the Pdgfrα signaling component pMek1/2 in *Shh* ^−/−^ ventricles (Figure S8). This is indicative of a downregulation of Pdgfrα signaling in *Shh*-negative ventricles.

**Figure 8:**
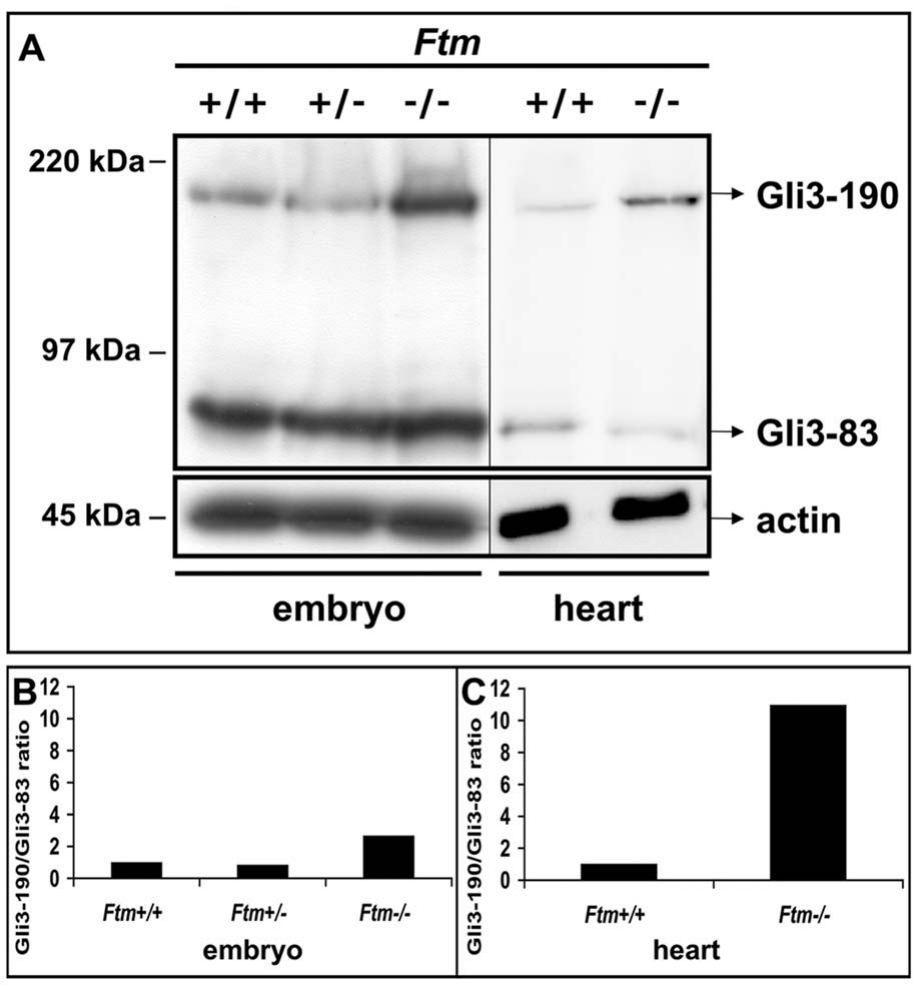
*Ftm*-negative hearts display a disturbance in Gli3 processing. (A) Western blot analysis of E11.5 embryo and heart protein lysates. Actin serves as loading control. In *Ftm*-negative embryos (n = 3), there is more Gli3-190 protein than in wild-type littermates, but an equal amount of Gli3-83. The amount of Gli3-190 protein is higher in *Ftm*-deficient than in wild-type hearts (n = 12, respectively), while conversely, there is less Gli3-83 in *Ftm* ^−/−^ hearts indicating a processing defect in *Ftm*-negative hearts. (B, C) Graphical evaluation of the Gli3-190/Gli3-83 ratio in wild-type and *Ftm*-deficient embryos and hearts, respectively. (B) The ratio of Gli3-190/Gli3-83 is 2.64 fold elevated in *Ftm*-homozygous mutant embryos. (C) Gli3-190/Gli3-83 ratio is 10.94 fold increased in *Ftm*-negative hearts.

Since the phenotype of *Shh*-deficient hearts, which display atrioventricular septal defects, appears to be much stronger than in *Ftm*-negative embryos [61], Ftm functions most likely downstream of Shh ligand in this pathway. The phenotypes of mice, which are negative for Ptc1 and Smo, two components of the Shh pathway downstream of its ligand, are also more severe than the *Ftm*-deficient phenotype [57], [62], so that we focused on the next players within this signaling cascade − the Gli proteins. We examined Gli3 processing by western blot analysis, using an antibody against the N-terminus of Gli3 that detects both the full-length (Gli3-190) and processed short, repressor (Gli3-83) forms. Previously, we were able to show that the ratio of Gli3-190/Gli3-83 is higher in *Ftm* ^−/−^ whole embryo protein lysates than in wild-type or *Ftm*-heterozygous ones (Figure 8A, B) [20]. In *Ftm*-deficient hearts, we also detected an increase of the Gli3-190/Gli-83 ratio at E11.5 (Figure 8A,C) confirming our assumption of a Gli3 processing defect. The ratio of Gli3-190/Gli3-83 in *Ftm*-negative embryos is 2.64 fold higher than in the wild-type (Figure 8B), while in *Ftm*-deficient hearts, the Gli3-190/Gli3-83 ratio is 10.94 fold higher than in their wild-type counterparts (Figure 8C).

#### Membranous ventricular septal defects in *Ftm*-negative mice are most likely not due to endocardial cushion defects

An interaction of inlet muscular VS and atrioventricular ECCs seems to be required for the beginning of membranous VS formation [8], [9]. Previously, it was suggested that defective ciliary function leads to a decreased cellularity of the ECCs [52]. So we looked for endocardial cushion morphology, the expression of marker genes and ECC proliferation (Figure S9). Morphologically, ECCs show a normal shape in *Ftm* ^−/−^ mice (Figure S9B) and also the marker gene expression of *Msh homeobox 1-like protein* (*Msx1*; Figure S9B) gives no hints indicating an abnormality in endocardial cushion development. Moreover, the number of proliferating ECCs is not altered in *Ftm*-deficient hearts at E11.5 (Figure S9C) and the atrioventricular valves exhibit a normal shape (data not shown). Since cilia are present on the surface of ECCs, it is interesting that the loss of Ftm does not seem to affect ECC proliferation. The proliferation in murine ventricles seems to be controlled mainly by Shh signals and so we examined ECC cilia-mediated signaling by analysing the ciliary localisation of Gli3. Interestingly, we did not detect Gli3 in or at wild-type ECC cilia (Figure S9D) indicating that these cilia do not transduce Shh signaling at all. Thus, although Ftm is present at the base of ECC cilia (Figure S9E), these cilia seem to be different from ventricular cilia as already suggested by the absence of acetylated α-tubulin (Figure 2D1). These data let suppose that the origin of perimembranous VSDs in *Ftm*-homozygous mutant hearts is not due to ECC dysfunction, but might be a defective outgrowth of the muscular VS.

## Discussion

### VSDs of *Ftm*-negative Mice are not only a Consequence of Impaired Left-right (LR) Asymmetry

Until now, the molecular mechanisms underlying VS development are largely unknown, but some factors have been elucidated which lead to the appearance of VSDs. One favoured reason for the occurrence of these congenital heart defects is the disturbancy of LR asymmetry. There is a high association between VSDs and LR asymmetry defects [63], [64]. Previously, we published that *Ftm*-deficient mice suffer from an impairment of LR asymmetry due to a dysfunction of nodal cilia [20]. This fact raises the possibility that the VSDs observed in the absence of Ftm are caused by randomized heart looping. 19% of *Ftm* ^−/−^ murine embryos display an abnormal heart looping [20], while 33% of these mice exhibit perimembranous VSDs. Thus, this laterality defect cannot be the exclusive reason for VSDs in *Ftm*-negative mice. Furthermore, other studies about hearts from embryos with abnormal LR development due to paralyzed node cilia show proper cardiac wall thickness [52], but 100% of all analyzed *Ftm* ^−/−^ embryos suffer from reduced wall thickness supporting evidence for other VSD-causing reasons.

Preliminarily, it was suggested that primary cilia in murine hearts contribute to proper cardiac development [52]. Since Ftm deficiency has been shown to result in ciliary dysfunction [20], [49], we examined in this study if *Ftm*-negative cardiac cilia cause perimembranous and muscular VSDs. The impact of Ftm absence on cardiac cilia is obvious, because *Ftm*-deficient cilia are shorter in atria and ventricles (Figure 3C). As an alteration of ciliary length gives a hint on a ciliary dysfunction, we investigated which molecular signals are mediated by cardiac cilia and if signaling is defective in the *Ftm*-negative state.

#### Do cardiac cilia mediate proliferative and hence muscular VS-generating signals?

We identified two signaling pathways which might be mediated by murine, ventricular cilia from E10.5 to E12.5, namely Shh and Pdgfrα signaling. The fact that atrial cilia do not transduce both pathways seems to be due to a difference in signal transduction of ventricular and atrial cells. Nevertheless, the correlation between ciliary presence and proliferation reduction as well as diminished wall thickness within the atria indicates that atrial cilia are associated with the proliferation of atrial cells at distinct regions. The control of this cilia-regulated atrial proliferation might be realized by mediating other signals than Shh or Pdgfrα signaling.

Our data let assume that there is a hierarchy between Shh and Pdgfrα signaling. In wild-type ventricles, Pdgfrα is located at cilia (Figure 6B). Since Pdgfrα is missing at *Shh*-negative, ventricular cilia (Figure 7D), Shh signaling seems to have an effect on Pdgfrα signaling in cardiac cilia of embryonic ventricles. In some cases, the loss of Pdgfrα alone already leads to VSDs in mice [65]. These findings provide the indication that the defect in *Ftm*-negative mice firstly seems to perturb Shh signaling and then secondly Pdgfrα signaling. Considering the heart phenotypes of *Shh*- [61], *Ptc1*- [62] and *Smo*-deficient mouse embryos [57], we suggest that the interruption in Shh signaling appears downstream of Smo, because the heart defects of these mutants are more severe than the cardiac phenotype of *Ftm* ^−/−^ embryos. Since the ratio of Gli3-190 to Gli3-83 is changed in *Ftm*-deficient embryonic hearts at E11.5 (Figure 8A, C), this could be the step in Shh signaling where the disturbance firstly takes place. The fact that not as much full-length Gli3 is cleaved to its shorter repressor form as in the wild-type implicates a defect in proteolytic processing of Gli3. Hence, *Ftm*-negative hearts display a higher amount of Gli3-190. Nevertheless, Shh signaling is downregulated in *Ftm*-deficient ventricles at E11.5 (Figure 5A). An explanation for this discrepancy could be a defect in the transformation of full-length Gli3 to its transcriptional activator form leading to a reduced activation of Shh target genes.

Since we measured a reduced expression of Shh and Pdgfrα target genes in E11.5 *Ftm*-deficient ventricles (Figure 5A) indicating a downregulation of both pathways and detected a reduction of proliferation at those regions of *Ftm* ^−/−^ ventricles where cilia are present, it is possible that cilia control ventricular proliferation by mediating Shh and Pdgfrα signals. We could not detect cardiac cilia on muscular VS cells (Figure 2A1i) and the proliferation rate of these cells is not significantly altered (Figure 4B1–B4, C). Hence, we suggest that VS formation is not based on cell proliferation in the apical region of the muscular VS.

Remarkably, ECC cilia which are clearly visible by detecting Arl13b (Figure 2D1) or detyrosinated tubulin (Figure 2E1i), but not by using an antibody against acetylated α-tubulin (Figure 2D1a), do not display the presence of Gli3 (Figure S9D) leading to the assumption that cilia of the ECCs do not mediate Shh or Pdgfrα signaling. Consequently, ECC proliferation is not significantly affected by Ftm deficiency. Since we did not find any morphological or molecular ECC alterations in *Ftm*-negative hearts (Figure S9), it is unlikely that defective ECCs are the reason for VSD appearance in *Ftm* ^−/−^ mice.

#### Reduced proliferation influences the thickness of cardiac walls and VS development

We detected a decrease in the thickness of atrial and ventricular walls at those positions where cilia previously acted (Figure 4D) in 100% of all *Ftm*-negative hearts. Since wall thickness is diminished in all cases, but the attenuation of muscular VS thickness occurs in 81.5% of all analysed *Ftm*-negative hearts and perimembranous VSDs only appear in 33% of *Ftm*-deficient hearts, the decline of the wall thickness seems to be the primary defect of cardiac ciliary dysfunction, while the VSD is a consequence of it. The entire phenotype of *Ftm*-negative mouse embryos is subject to a variation reaching from embryos with extrinsically mild defects to severly deformed embryos. Nevertheless, all *Ftm* ^−/−^ embryos die at latest around birth. The reason for the phenotype variation is unknown, but maybe, it is the same, which causes differences in VS development of *Ftm*-deficient hearts. Potentially, the number of functional cilia plays a decisive role in the phenotype variation. If there is a threshold of cilia-mediated signals determing the severity of the mutant phenotype, it could be possible that the fewer cilia are present the stronger shapes the phenotype.

Interestingly, the percentage of perimembranous VSDs is higher in *Ftm* ^−/−^ hearts at E13.5 (40%) (Figure S1B) than at E17.5 (22%) (Figure S1N) suggesting that the defective VS development in the absence of Ftm occurs due to a developmental delay. Another explanation is that mice displaying a stronger phenotype and therefore a perimembranous VSD die earlier within the embryonic development than those with a milder phenotype. The second possibility is supported by the following facts: It is obvious that lethality at early embryonic stages (e.g. E13.5) takes place when Ftm is missing. *Ftm*-deficient embryos which suffer from multiple defects die earlier than those displaying a milder phenotype. Consequently, we observe exclusively milder mutant phenotypes at late embryonic stages (Table S1). Remarkably, some *Ftm*-negative embryos at late embryonic days which show mild mutant phenotypes suffer from perimembranous VSDs meaning that most organs of these embryos develop properly and indicating that a possible developmental delay only affects heart development. This argues clearly against a developmental delay. Moreover, muscular VSDs are detected in a high frequency at late embryonic days like E17.5 (89%) (Figure S1O) demonstrating that the observed VSDs are hardly based on a developmental delay.

The analysis of wall thickness in *Ftm*-negative hearts results in a clear subdivision appearing in the atria. We observed thinner walls where cilia had been present and normal wall thickness at those sites which never showed any cilia. However, ventricular walls display reduced wall thickness at both ciliary and non-ciliary regions (Figure 4D). It is known that the muscular VS consists of cardiomyocytes with both left-ventricular and right-ventricular identities [11] indicating that the ciliary dysfunction leads to a decrease of ventricular proliferation and hence to the appearance of VSDs. In contrary to ventricular development, atrial septal formation seems to be independent of ciliary function, because the atrial septum appears to be unaffected in *Ftm*-negative embryos.

#### Model of VS formation

**Figure 9:**
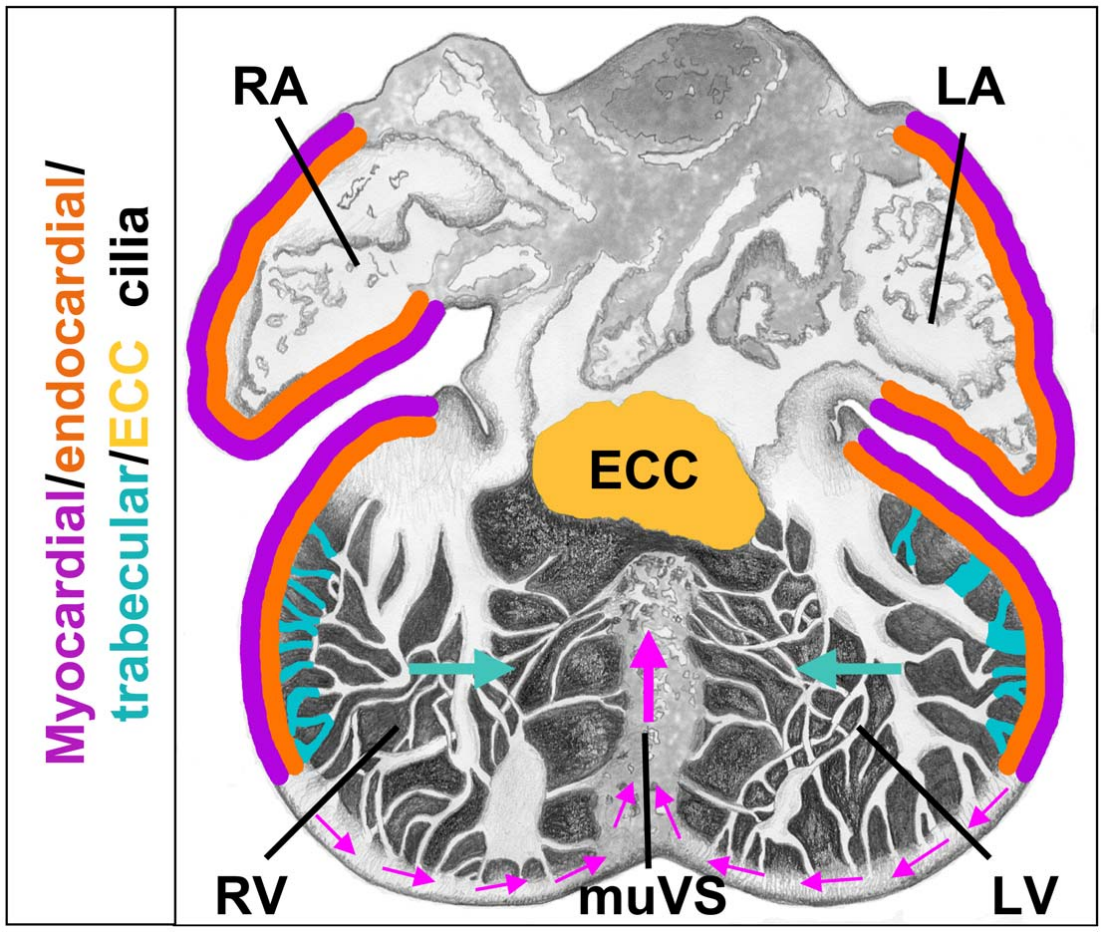
Model of VS development. Myocardial (violett), endocardial (orange) and most likely trabecular cilia (turquoise) regulate proliferation at distinct cardiac regions. In the ventricles, the cell proliferation in these regions results in wall thickness control, trabecular formation and a push of cells toward the base of the muscular ventricular septum (indicated by pink arrows). ECC cilia (yellow) seem to be different from the other cardiac cilia, since they do not regulate ECC proliferation. LA, left atrium; RA, right atrium; LV, left ventricle; RV, right ventricle; muVS, muscular ventricular septum.

Assuming that cardiac cilia regulate proliferation, our data allow us to propose a model for how the VS is generated. Ventricular cells at distinct positions assemble monocilia on their surface. These cardiac cilia contain components of Shh and Pdgfrα signaling most likely permitting them to mediate those signals. Thus, target genes of those signaling pathways are activated in cilia-possessing, ventricular cells. Interestingly, in ventricular cilia Pdgfrα signaling acts downstream of Shh signaling. In the end, the mediation of these different signals by cardiac cilia stimulates the cells to proliferate and this proliferation leads to a push of cells to the base of the muscular VS (Figure 9). Thus, in *Ftm*-negative ventricles the wall thickness of non-ciliary regions near the base of the muscular VS is significantly thinner (Figure 4D) due to the numeral reduction of cells which are pushed towards the base of the muscular VS. Both, the pushed cells and the trabecular formations shape the muscular VS which on its part grows to a certain point and then interacts molecularly with the ECCs. In turn, the ECCs start to shape the membranous VS which then grows towards the muscular VS. When they meet, they fuse and the development of the VS is finished. So finally, the muscular VS consists of cells which descend from the left and right ventricular walls and from the trabecular formations. Thus, our model supports the idea of muscular septal formation as a product of a passive process based on proliferation of cells at distinct regions in the left and right ventricles.

## Supporting Information

Figure S1 **Defects of VS development in** ***Ftm*** **-negative mice are most likely not due to a developmental delay.** Septum length and width was measured as well as the percentage of murine hearts suffering from perimembranous and muscular VSDs was determined at E13.5 (A, B, C), at E14.5 (D, E, F), at E15.5 (G, H, I), at E16.5 (J, K, L) and at E17.5 (M, N, O). (A, D, G, J, M) Septum measurements of wild-type and *Ftm*-deficient ventricles at E13.5 (A), E14.5 (D), E15.5 (G), E16.5 (J) and E17.5 (M). Septum width was measured at different levels of the VS – apical, medial and basal. The results of all levels together were used to compile statistics. (A) At E13.5, *Ftm*-negative VS (n = 5) are significantly thinner (p = 0.017) than their wild-type counterparts (n = 5), while the length of *Ftm* ^−/−^ VS (n = 5) is not significantly altered in comparison to the wild-type ones (n = 5). (D) At E14.5, *Ftm*-negative VS (n = 6) are significantly thinner (p = 0.003) than their wild-type counterparts (n = 6), while the length of *Ftm* ^−/−^ VS (n = 6) is not significantly altered in comparison to the wild-type ones (n = 6). (G) At E15.5, *Ftm*-negative VS (n = 3) are significantly thinner (p = 0.046) than their wild-type counterparts (n = 3), while the length of *Ftm* ^−/−^ VS (n = 3) is not significantly altered in comparison to the wild-type ones (n = 3). (J) At E16.5, *Ftm*-negative VS (n = 4) are significantly thinner (p = 0.007) than their wild-type counterparts (n = 4), while the length of *Ftm* ^−/−^ VS (n = 4) is not significantly altered in comparison to the wild-type ones (n = 4). (M) At E17.5, *Ftm*-negative VS (n = 9) are significantly thinner (p = 0.003) than their wild-type counterparts (n = 5), while the length of *Ftm* ^−/−^ VS (n = 9) is not significantly altered in comparison to the wild-type ones (n = 5). Percentages of hearts affected by perimembranous or muscular VSDs were calculated from the very same number of embryos used in A, D, G, J and M. None of the wild-type embryos displays a VSD. (B) At E13.5, 40% of all analyzed *Ftm*-deficient embryos exhibit a perimembranous VSD, (E) at E14.5 50%, (H) at E15.5 67%, (K) at E16.5 0% and at E17.5 22%. (C) At E13.5, 80% of all analyzed *Ftm*-deficient embryos show a muscular VSD, (E) at E14.5 83%, (H) at E15.5 100%, (K) at E16.5 50% and at E17.5 89%. VS, ventricular septum; VSD, ventricular septal defect.
(TIF)

Figure S2 **Cilia are not present on VS cells.** Immunofluorescence on transverse heart sections at E12.5. Cilia are stained in red by marking Arl13b and cell nuclei in blue by the use of DAPI. Scale bar (in white) represents a length of 100 µm. ECCs are encircled by a yellow line, VS cells by a green line. White arrowheads point to cilia which are present on trabecular cells, but not on VS cells.
(TIF)

Figure S3 **Primary cilia are present on myocardial and endocardial cells.** (A–C) Immunohistochemistry on transverse heart sections at E11.5. Cilia are stained in green by acetylated α-tubulin and cell nuclei in blue by DAPI. Scale bars (in white) represent a length of 2 µm. (A–C) White arrows point to cilia. (A, B) Myocardial cells (A; red staining; marked by tropomyosin) and endocardial cells (B; red staining; marked by ErbB3) possess cilia. (C) Cardiac fibroblasts (red staining; marked by DDR2) do not show any cilia. (D) Schematic illustration of ciliary distribution in embryonic mouse hearts. We found cilia at E10.5–12.5 on myocardial cells (violett), endocardial cells (orange), ECCs (yellow) and trabecles (turquoise). LA, left atrium; RA, right atrium; LV, left ventricle; RV, right ventricle; muVS, muscular ventricular septum.
(TIF)

Figure S4 **Co-localisation of Ftm with the basal body and centrosome marker γ-tubulin.** (A, B) Immunohistochemistry on transverse heart sections at E11.5. Centrosomes/basal bodies are marked in green by γ-tubulin and cell nuclei in blue by DAPI. Scale bars (in white) represent a length of 2 µm. (A) Ftm staining (red) partially overlaps with the staining of the centrosome/basal body (green). (B) In *Ftm*-negative hearts, Ftm is missing at the centrosome/basal body of cilia.
(TIF)

Figure S5 **Apoptosis at E11.5 and proliferation rate at E14.5 is unaltered in** ***Ftm*** **-deficient hearts.** (A) Apoptosis studies by TUNEL stainings in E11.5 hearts. No significant differences can be detected in wild-type (n = 3), *Ftm*-heterozygous mutant (n = 3) and *Ftm*-homozygous mutant (n = 3) heart compartments. (B) Proliferation rate is determined by the relation of dividing (BrdU-marked) cells to the number of all cells in distinct heart regions at E14.5 (*Ftm* ^+/+^: n = 3 hearts; *Ftm* ^−/−^: n = 3 hearts). In none of the investigated *Ftm*-negative heart compartments, cell proliferation is significantly altered.
(TIF)

Figure S6 **Expression alterations of genes involved in cell cycle progression and proliferation in atria and ventricles.** Semi-quantitative PCR analysis of wild-type and *Ftm* ^−/−^ atrial and ventricular tissue at E11.5. *Hprt* serves as loading control. Expression of *cyclin E* is downregulated and expression of *p27* is upregulated in *Ftm*-negative atria and ventricles suggesting a disturbance in cell cycle progression and proliferation.
(TIF)

Figure S7 **Gli3-190 localizes at the base of ventricular cilia.** Immunohistochemistry on transverse heart sections at E11.5. (A) Centrosomes/basal bodies are marked in green by γ-tubulin and cell nuclei in blue by DAPI. Scale bar (in white) represents a length of 2 µm. Gli3-190 staining (red) partially overlaps with the staining of the centrosome/basal body (green). (B) Pericentriolar material at the base of cilia is stained in blue by pericentrin and the ciliary axoneme in green by acetylated α-tubulin. Gli3-190 (red staining) co-localizes with pericentrin and hence is present at the base of ventricular cilia.
(TIF)

Figure S8 **pMek1/2, a Pdgfrα signaling pathway component, is downregulated in** ***Shh*** **-negative ventricles.** Western blot analysis of E11.5 ventricular protein lysates. Gapdh serves as loading control. In *Shh*-negative ventricles (n = 3), there is less phosphorylated Mek1/2 protein than in wild-type littermates.
(TIF)

Figure S9 **Endocardial cushion development is not altered in** ***Ftm*** **-negative embryos.** (A, B) In situ hybridizations on heart sections at E11.5. Endocardial cushion marker expression of *Msx1* is unchanged in *Ftm*-deficient, murine hearts (compare inlets in A and B). (C) Proliferation rate is determined by the relation of dividing (BrdU-marked) ECCs to the number of all ECCs in this region at E11.5 (*Ftm* ^+/+^: n = 3 hearts; *Ftm* ^−/−^: n = 3 hearts). The number of proliferating ECCs is not significantly altered in *Ftm*-negative hearts. (D, E) Immunofluorescence on transverse heart sections at E11.5. ECC cilia are marked in red by Arl13b. Scale bars (in white) represent a length of 2 µm. (D) Gli3 (green) is missing at ECC cilia. (E) Ftm (green) is present at ECC cilia.
(TIF)

Table S1 **Phenotypes of all analyzed** ***Ftm*** **-negative embryos.** The phenotypes of all analyzed *Ftm*-negative embryos in the developmental stages E13.5 to E17.5 is depicted in this table. The “x” symbolizes the appearance of the defect. pVSD, perimembranous ventricular septal defect; mVSD, muscular ventricular septal defect.
(DOC)
