# Supplementary figures and images for: The Ciliary Protein Ftm Is Required for Ventricular Wall and Septal Development
Source: PLoS One. 2013 Feb 28;8(2):e57545. doi: 10.1371/journal.pone.0057545 (PMC3585374; doi:10.1371/journal.pone.0057545)

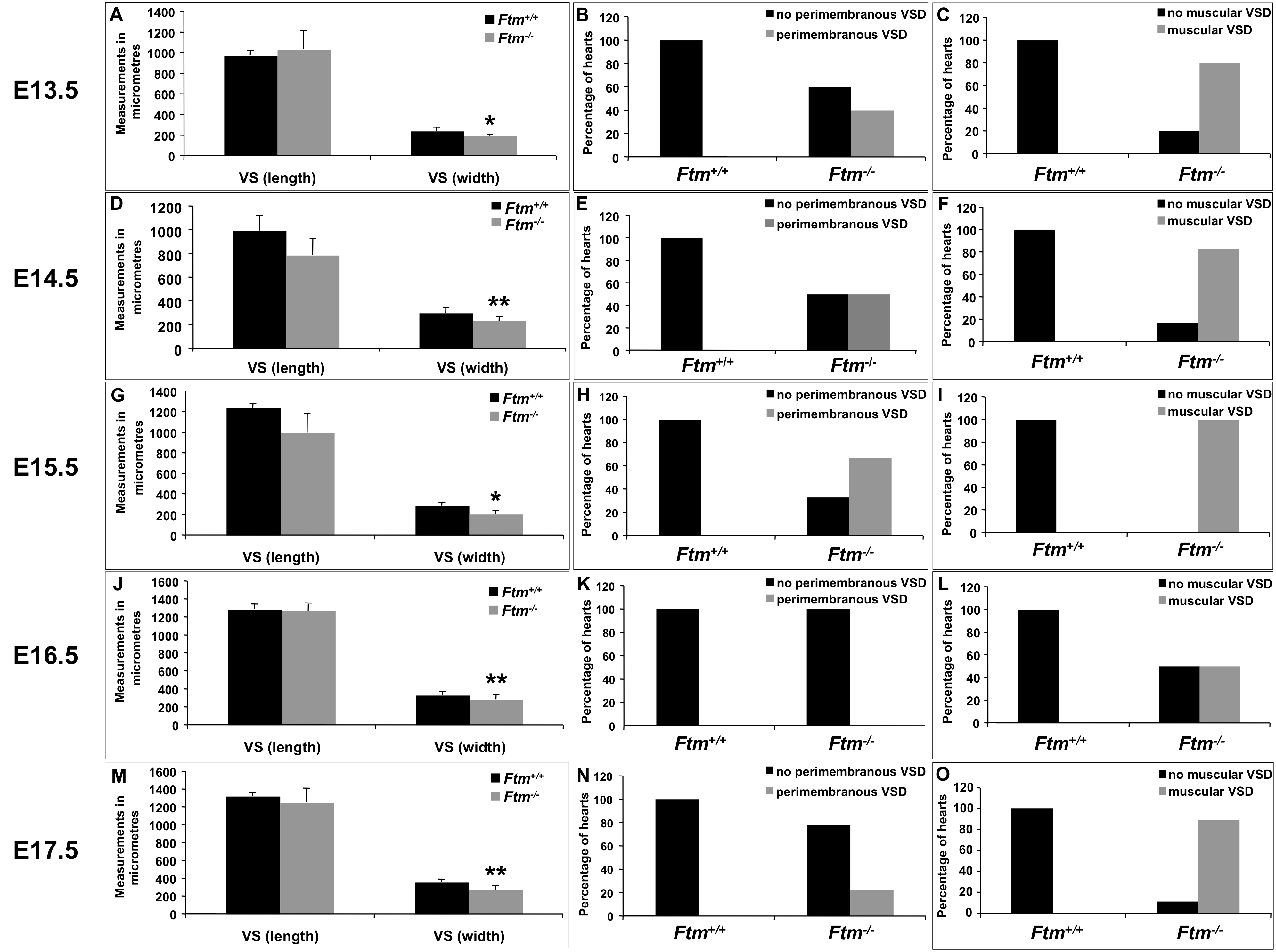

Supplement: Figure S1 — Defects of VS development in Ftm -negative mice are most likely not due to a developmental delay. Septum length and width was measured as well as the percentage of murine hearts suffering from perimembranous and muscular VSDs was determined at E13.5 (A, B, C), at E14.5 (D, E, F), at E15.5 (G, H, I), at E16.5 (J, K, L) and at E17.5 (M, N, O). (A, D, G, J, M) Septum measurements of wild-type and Ftm-deficient ventricles at E13.5 (A), E14.5 (D), E15.5 (G), E16.5 (J) and E17.5 (M). Septum width was measured at different levels of the VS – apical, medial and basal. The results of all levels together were used to compile statistics. (A) At E13.5, Ftm-negative VS (n = 5) are significantly thinner (p = 0.017) than their wild-type counterparts (n = 5), while the length of Ftm −/− VS (n = 5) is not significantly altered in comparison to the wild-type ones (n = 5). (D) At E14.5, Ftm-negative VS (n = 6) are significantly thinner (p = 0.003) than their wild-type counterparts (n = 6), while the length of Ftm −/− VS (n = 6) is not significantly altered in comparison to the wild-type ones (n = 6). (G) At E15.5, Ftm-negative VS (n = 3) are significantly thinner (p = 0.046) than their wild-type counterparts (n = 3), while the length of Ftm −/− VS (n = 3) is not significantly altered in comparison to the wild-type ones (n = 3). (J) At E16.5, Ftm-negative VS (n = 4) are significantly thinner (p = 0.007) than their wild-type counterparts (n = 4), while the length of Ftm −/− VS (n = 4) is not significantly altered in comparison to the wild-type ones (n = 4). (M) At E17.5, Ftm-negative VS (n = 9) are significantly thinner (p = 0.003) than their wild-type counterparts (n = 5), while the length of Ftm −/− VS (n = 9) is not significantly altered in comparison to the wild-type ones (n = 5). Percentages of hearts affected by perimembranous or muscular VSDs were calculated from the very same number of embryos used in A, D, G, J and M. None of the wild-type embryos displays a VSD. (B) At E13.5, [file pone.0057545.s001.tif]

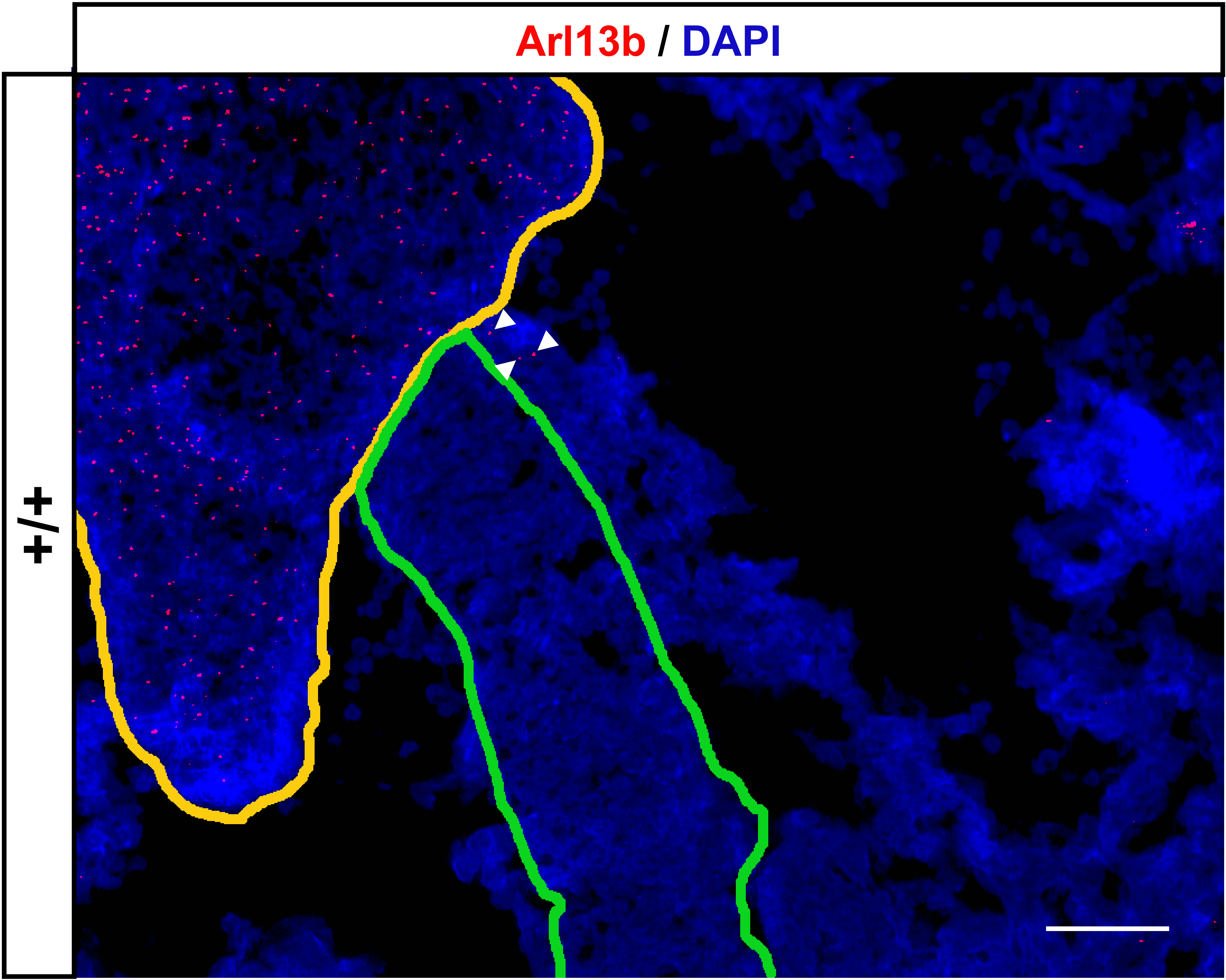

Supplement: Figure S2 — Cilia are not present on VS cells. Immunofluorescence on transverse heart sections at E12.5. Cilia are stained in red by marking Arl13b and cell nuclei in blue by the use of DAPI. Scale bar (in white) represents a length of 100 µm. ECCs are encircled by a yellow line, VS cells by a green line. White arrowheads point to cilia which are present on trabecular cells, but not on VS cells. (TIF) [file pone.0057545.s002.tif]

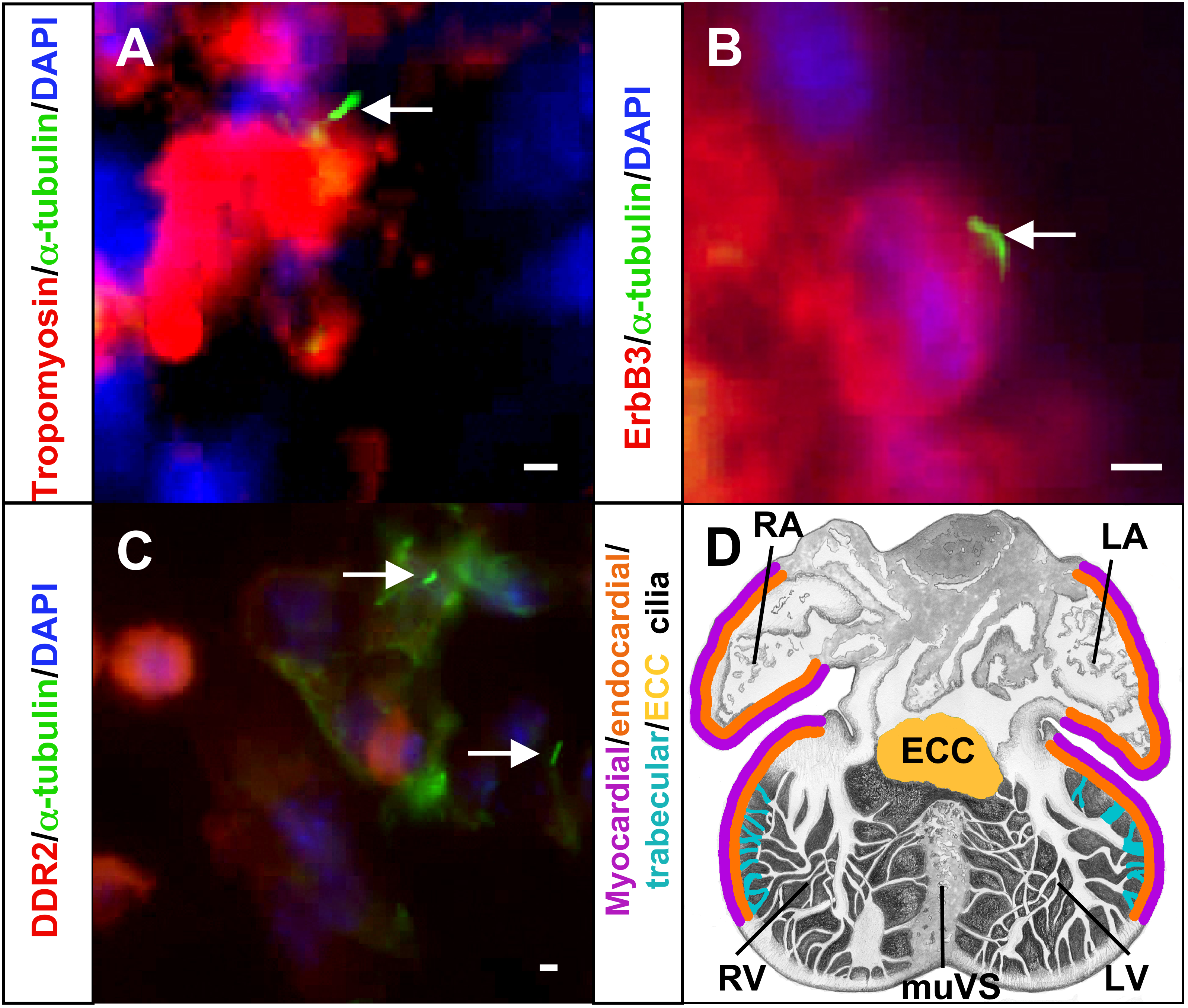

Supplement: Figure S3 — Primary cilia are present on myocardial and endocardial cells. (A–C) Immunohistochemistry on transverse heart sections at E11.5. Cilia are stained in green by acetylated α-tubulin and cell nuclei in blue by DAPI. Scale bars (in white) represent a length of 2 µm. (A–C) White arrows point to cilia. (A, B) Myocardial cells (A; red staining; marked by tropomyosin) and endocardial cells (B; red staining; marked by ErbB3) possess cilia. (C) Cardiac fibroblasts (red staining; marked by DDR2) do not show any cilia. (D) Schematic illustration of ciliary distribution in embryonic mouse hearts. We found cilia at E10.5–12.5 on myocardial cells (violett), endocardial cells (orange), ECCs (yellow) and trabecles (turquoise). LA, left atrium; RA, right atrium; LV, left ventricle; RV, right ventricle; muVS, muscular ventricular septum. (TIF) [file pone.0057545.s003.tif]

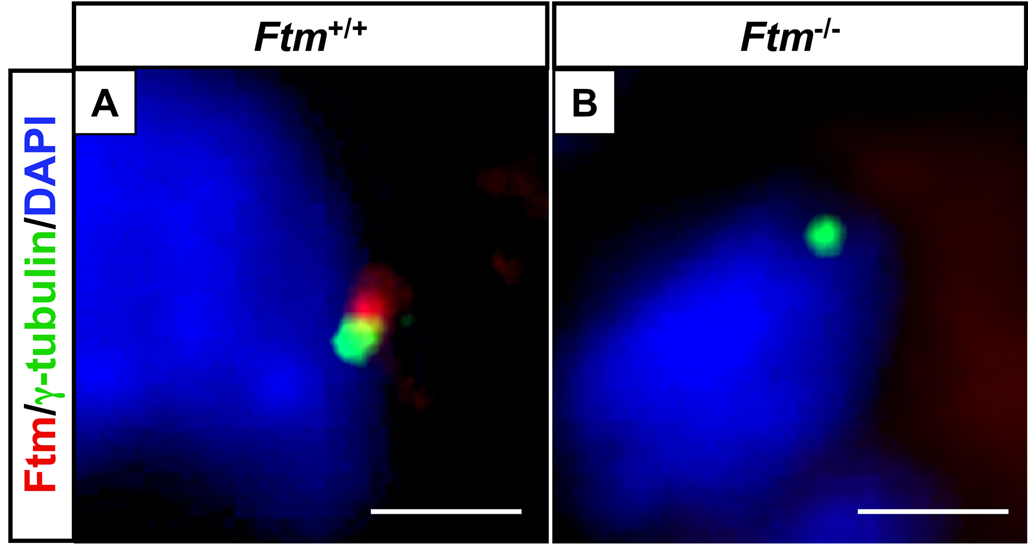

Supplement: Figure S4 — Co-localisation of Ftm with the basal body and centrosome marker γ-tubulin. (A, B) Immunohistochemistry on transverse heart sections at E11.5. Centrosomes/basal bodies are marked in green by γ-tubulin and cell nuclei in blue by DAPI. Scale bars (in white) represent a length of 2 µm. (A) Ftm staining (red) partially overlaps with the staining of the centrosome/basal body (green). (B) In Ftm-negative hearts, Ftm is missing at the centrosome/basal body of cilia. (TIF) [file pone.0057545.s004.tif]

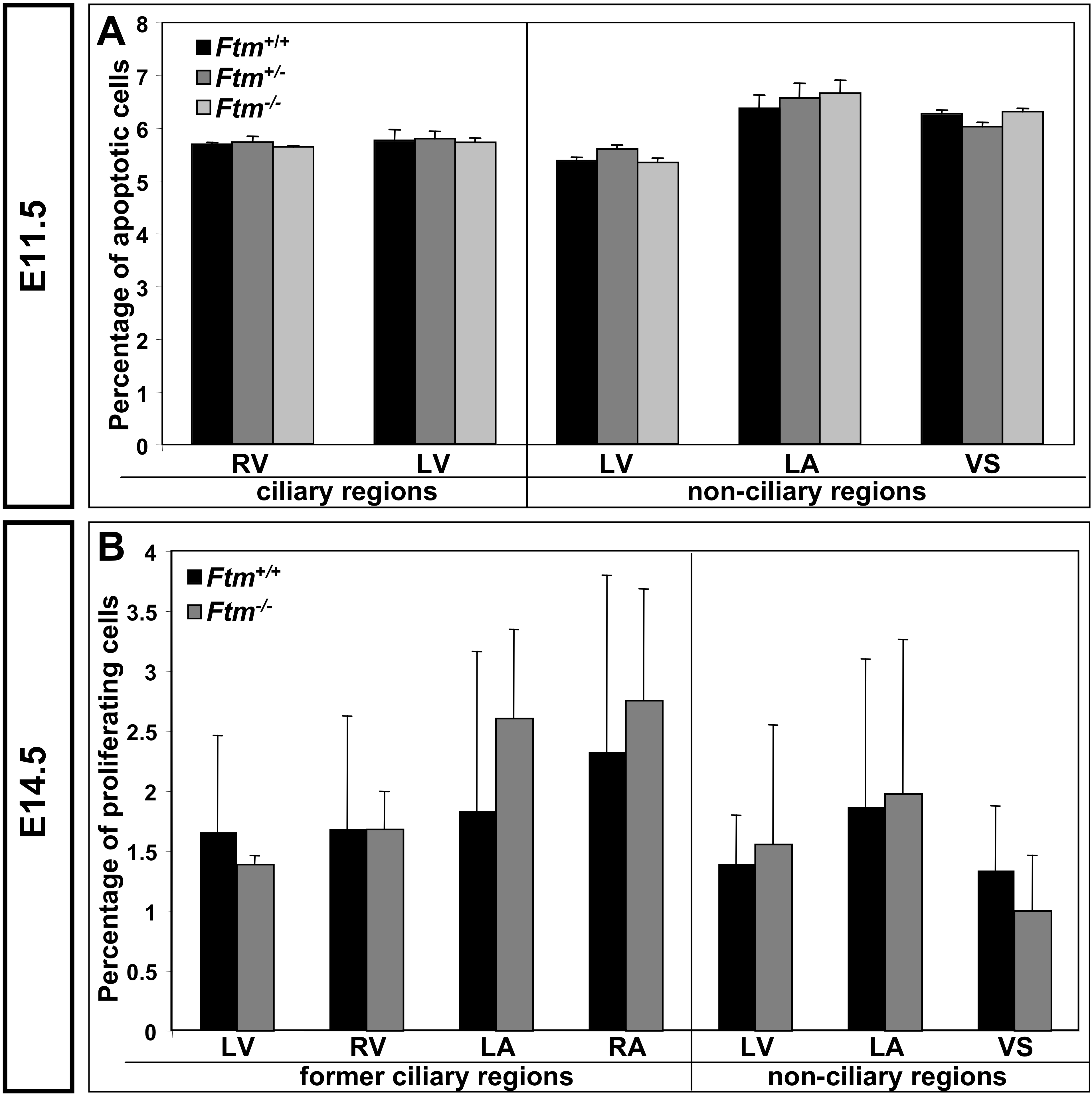

Supplement: Figure S5 — Apoptosis at E11.5 and proliferation rate at E14.5 is unaltered in Ftm -deficient hearts. (A) Apoptosis studies by TUNEL stainings in E11.5 hearts. No significant differences can be detected in wild-type (n = 3), Ftm-heterozygous mutant (n = 3) and Ftm-homozygous mutant (n = 3) heart compartments. (B) Proliferation rate is determined by the relation of dividing (BrdU-marked) cells to the number of all cells in distinct heart regions at E14.5 (Ftm +/+: n = 3 hearts; Ftm −/−: n = 3 hearts). In none of the investigated Ftm-negative heart compartments, cell proliferation is significantly altered. (TIF) [file pone.0057545.s005.tif]

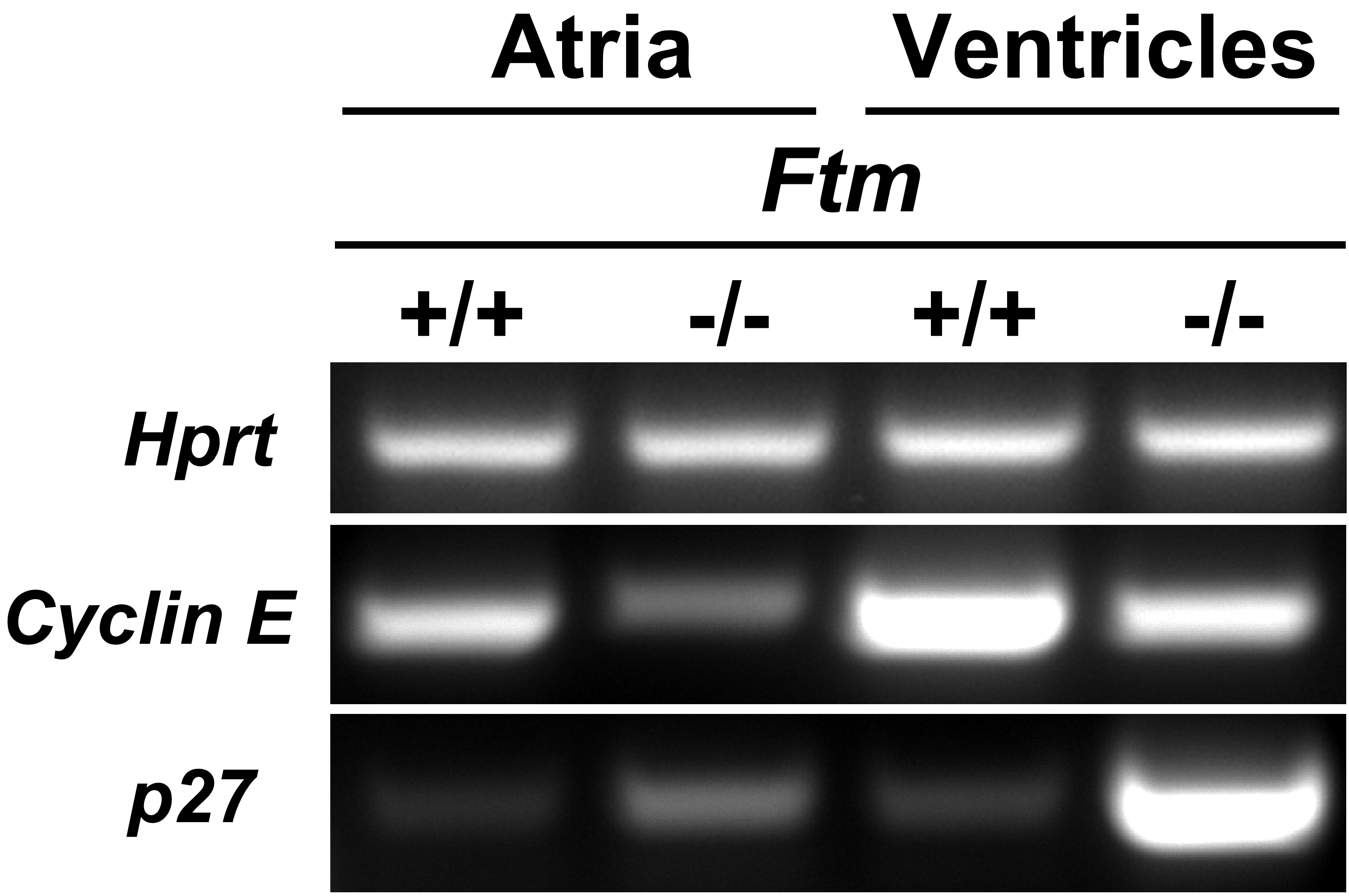

Supplement: Figure S6 — Expression alterations of genes involved in cell cycle progression and proliferation in atria and ventricles. Semi-quantitative PCR analysis of wild-type and Ftm −/− atrial and ventricular tissue at E11.5. Hprt serves as loading control. Expression of cyclin E is downregulated and expression of p27 is upregulated in Ftm-negative atria and ventricles suggesting a disturbance in cell cycle progression and proliferation. (TIF) [file pone.0057545.s006.tif]

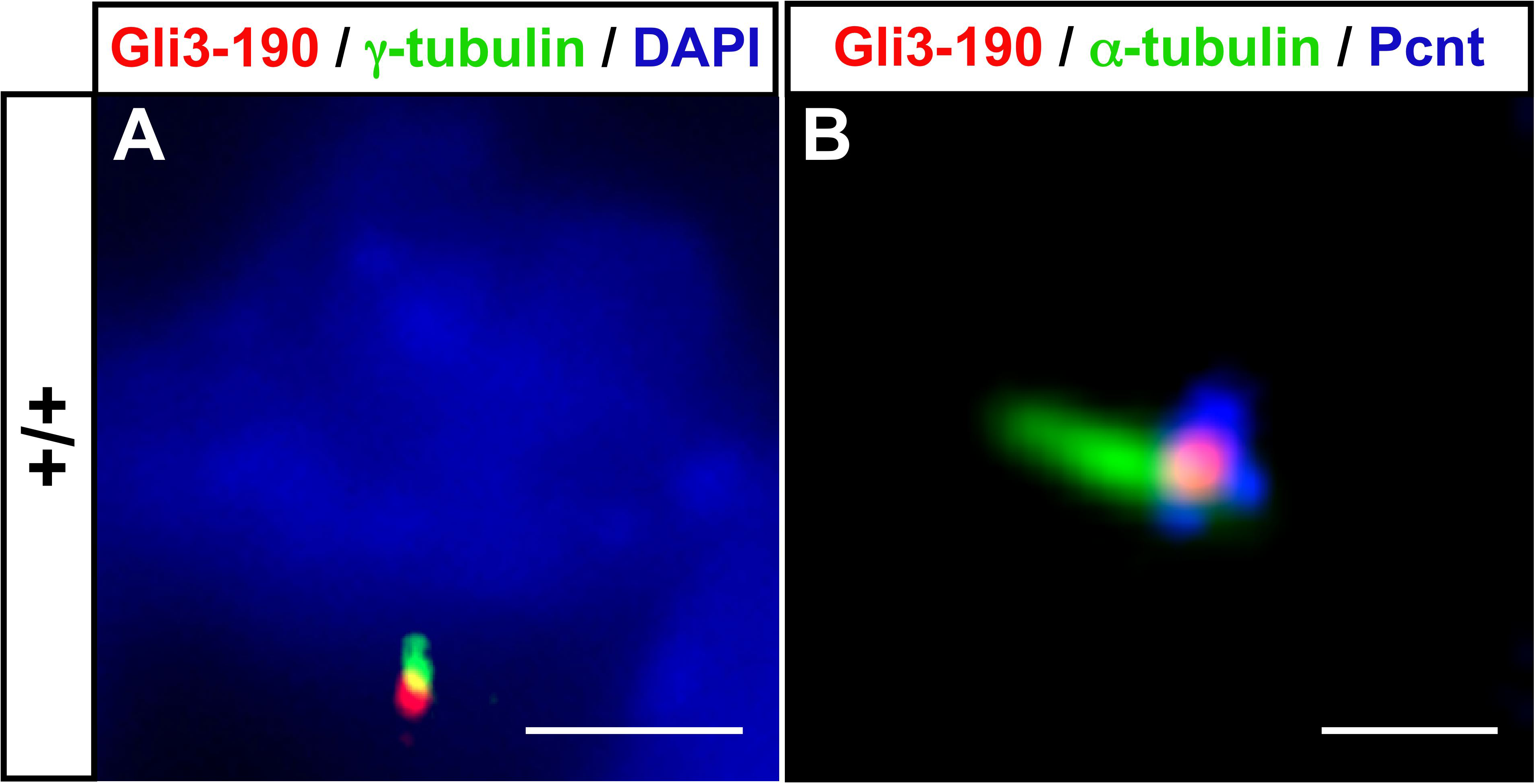

Supplement: Figure S7 — Gli3-190 localizes at the base of ventricular cilia. Immunohistochemistry on transverse heart sections at E11.5. (A) Centrosomes/basal bodies are marked in green by γ-tubulin and cell nuclei in blue by DAPI. Scale bar (in white) represents a length of 2 µm. Gli3-190 staining (red) partially overlaps with the staining of the centrosome/basal body (green). (B) Pericentriolar material at the base of cilia is stained in blue by pericentrin and the ciliary axoneme in green by acetylated α-tubulin. Gli3-190 (red staining) co-localizes with pericentrin and hence is present at the base of ventricular cilia. (TIF) [file pone.0057545.s007.tif]

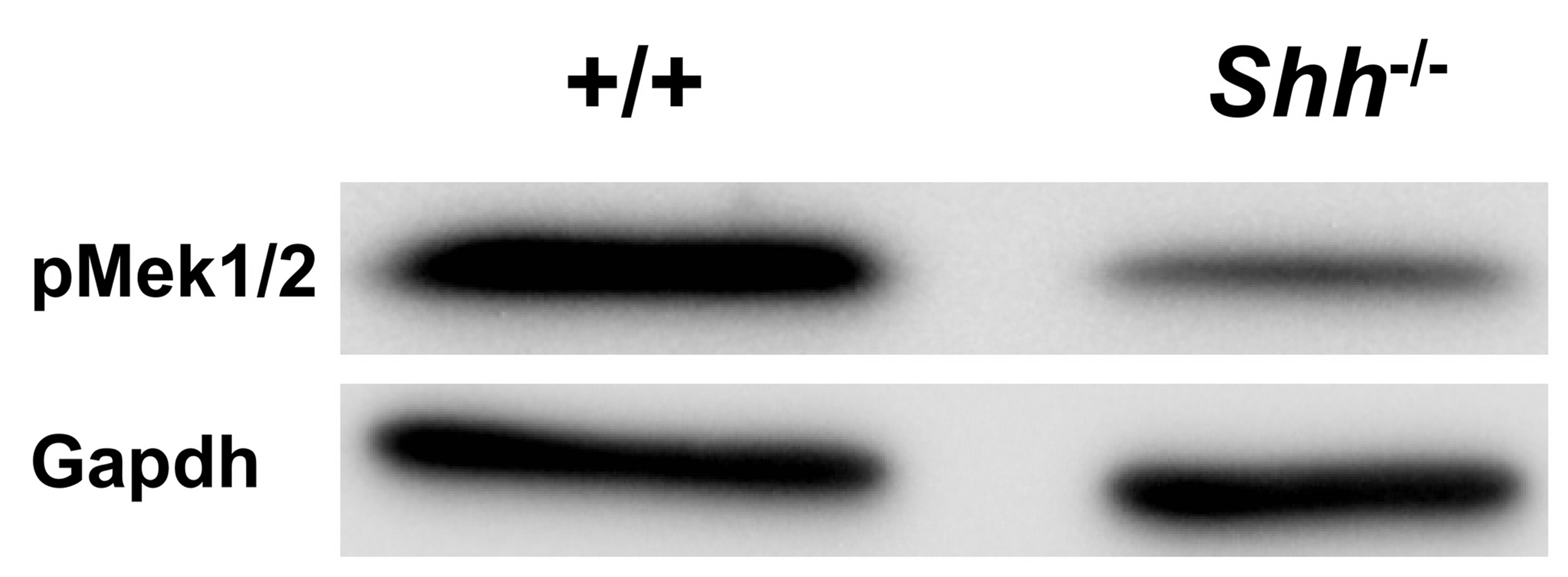

Supplement: Figure S8 — pMek1/2, a Pdgfrα signaling pathway component, is downregulated in Shh -negative ventricles. Western blot analysis of E11.5 ventricular protein lysates. Gapdh serves as loading control. In Shh-negative ventricles (n = 3), there is less phosphorylated Mek1/2 protein than in wild-type littermates. (TIF) [file pone.0057545.s008.tif]

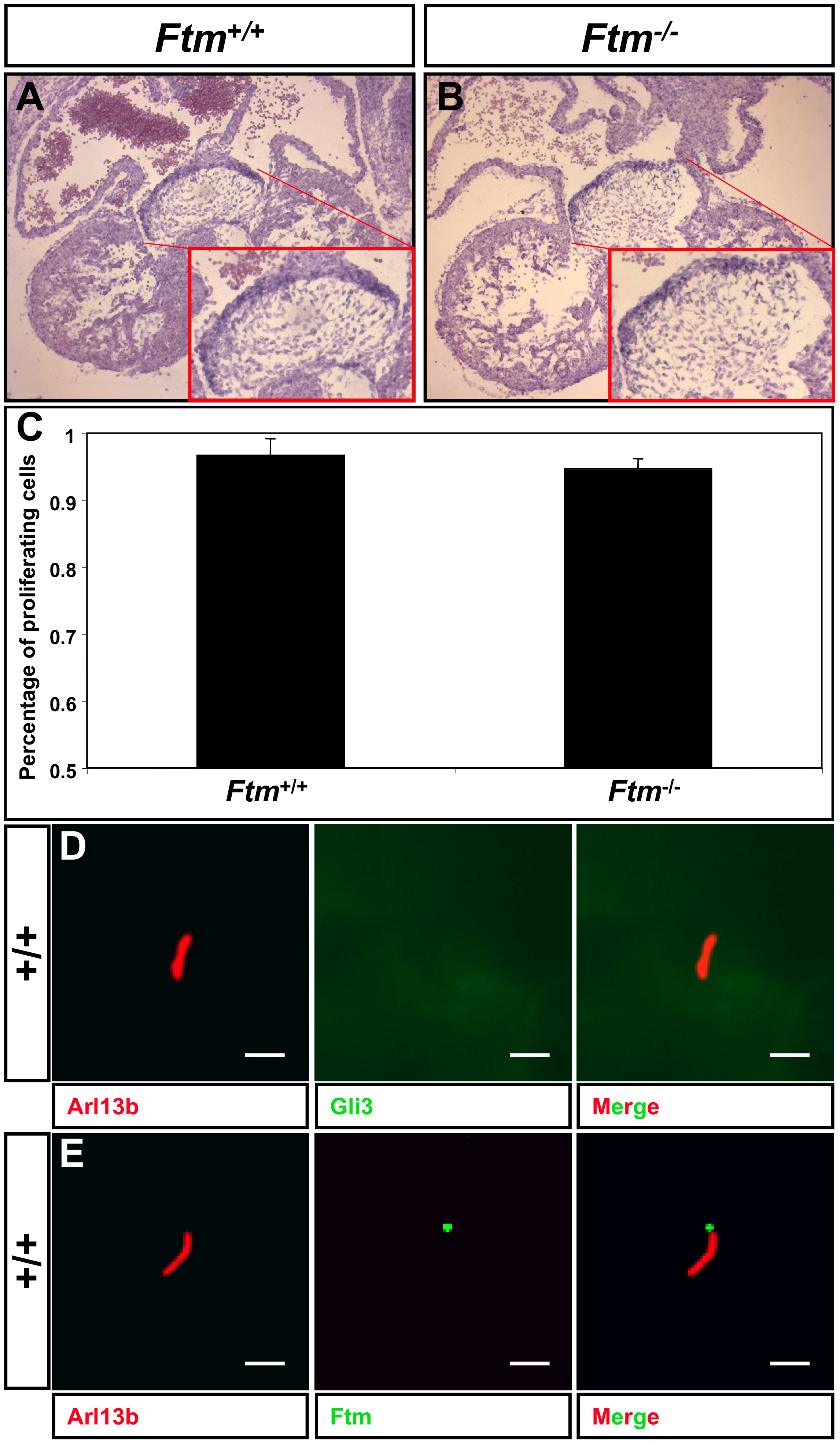

Supplement: Figure S9 — Endocardial cushion development is not altered in Ftm -negative embryos. (A, B) In situ hybridizations on heart sections at E11.5. Endocardial cushion marker expression of Msx1 is unchanged in Ftm-deficient, murine hearts (compare inlets in A and B). (C) Proliferation rate is determined by the relation of dividing (BrdU-marked) ECCs to the number of all ECCs in this region at E11.5 (Ftm +/+: n = 3 hearts; Ftm −/−: n = 3 hearts). The number of proliferating ECCs is not significantly altered in Ftm-negative hearts. (D, E) Immunofluorescence on transverse heart sections at E11.5. ECC cilia are marked in red by Arl13b. Scale bars (in white) represent a length of 2 µm. (D) Gli3 (green) is missing at ECC cilia. (E) Ftm (green) is present at ECC cilia. (TIF) [file pone.0057545.s009.tif]
