# Supplementary material for: The Ciliary Protein Ftm Is Required for Ventricular Wall and Septal Development
Source: PLoS One. 2013 Feb 28;8(2):e57545. doi: 10.1371/journal.pone.0057545 (PMC3585374; doi:10.1371/journal.pone.0057545)
Supplement: Table S1 — Phenotypes of all analyzed Ftm -negative embryos. The phenotypes of all analyzed Ftm-negative embryos in the developmental stages E13.5 to E17.5 is depicted in this table. The “x” symbolizes the appearance of the defect. pVSD, perimembranous ventricular septal defect; mVSD, muscular ventricular septal defect. (DOC) [file pone.0057545.s010.doc]

|  | **pVSD*** | **mVSD^†^** | **Laterality defects** | **Polydactyly** | **Exencephaly** | **Microphthalmia** | **Kidney cysts** | **Liver cysts** |
| --- | --- | --- | --- | --- | --- | --- | --- | --- |
| **E13.5** |  |  |  |  |  |  |  |  |
| *Ftm*^-/-^ |  |  |  |  |  |  |  |  |
|  |  |  |  |  |  |  |  |  |
| Embryo1 |  | x | x | x | x |  |  |  |
| Embryo2 | x | x | x | x | x | x |  |  |
| Embryo3 | x | x |  | x |  |  |  |  |
| Embryo4 |  |  |  |  |  |  |  |  |
| Embryo5 |  | x |  |  |  | x |  |  |
|  |  |  |  |  |  |  |  |  |
|  |  |  |  |  |  |  |  |  |
| **E14.5** |  |  |  |  |  |  |  |  |
| *Ftm*^-/-^ |  |  |  |  |  |  |  |  |
|  |  |  |  |  |  |  |  |  |
| Embryo1 | x | x | x | x |  | x |  |  |
| Embryo2 |  | x | x |  | x |  |  |  |
| Embryo3 |  | x |  | x |  |  |  |  |
| Embryo4 |  |  |  |  |  |  |  |  |
| Embryo5 | x | x |  |  |  |  |  |  |
| Embryo6 | x | x |  | x | x |  |  |  |
|  |  |  |  |  |  |  |  |  |
|  |  |  |  |  |  |  |  |  |
| **E15.5** |  |  |  |  |  |  |  |  |
| *Ftm*^-/-^ |  |  |  |  |  |  |  |  |
|  |  |  |  |  |  |  |  |  |
| Embryo1 | x | x |  | x |  |  |  |  |
| Embryo2 |  | x |  |  |  | x |  |  |
| Embryo3 | x | x |  |  |  |  |  |  |
|  |  |  |  |  |  |  |  |  |
|  |  |  |  |  |  |  |  |  |
| **E16.5** |  |  |  |  |  |  |  |  |
| *Ftm*^-/-^ |  |  |  |  |  |  |  |  |
|  |  |  |  |  |  |  |  |  |
| Embryo1 |  | x |  | x |  |  |  |  |
| Embryo2 |  |  |  |  |  |  |  |  |
| Embryo3 |  |  |  |  |  |  |  |  |
| Embryo4 |  | x |  |  |  |  |  |  |
|  |  |  |  |  |  |  |  |  |
|  |  |  |  |  |  |  |  |  |
| **E17.5** |  |  |  |  |  |  |  |  |
| *Ftm*^-/-^ |  |  |  |  |  |  |  |  |
|  |  |  |  |  |  |  |  |  |
| Embryo1 | x | x |  |  |  |  |  |  |
| Embryo2 |  | x |  | x |  |  |  |  |
| Embryo3 |  | x |  |  |  |  |  |  |
| Embryo4 |  | x |  |  |  |  |  |  |
| Embryo5 |  |  |  |  |  |  |  |  |
| Embryo6 |  | x |  |  |  | x | x | x |
| Embryo7 |  | x |  |  |  |  |  |  |
| Embryo8 | x | x |  | x |  |  | x | x |
| Embryo9 |  | x |  |  |  |  |  |  |
|  |  |  |  |  |  |  |  |  |
